# Association of Socioeconomic Factors With Oral Health in Older Adults: A Systematic Review and Meta‐Analysis

**DOI:** 10.1111/ger.70079

**Published:** 2026-05-15

**Authors:** Saif S. Syed, Sungkrit Pojmonpiti, Barbara Janssens, Murali Srinivasan, Frauke Müller, Georgios Tsakos

**Affiliations:** ^1^ Department of Epidemiology and Public Health University College London London UK; ^2^ Institute of Dentistry Queen Mary University of London London UK; ^3^ ELOHA (Equal Lifelong Oral Health for All) Research Group, Department of Oral Health Sciences, Faculty of Medicine and Health Sciences University of Ghent Ghent Belgium; ^4^ Department of Neurology Ghent University Hospital Ghent Belgium; ^5^ Dental Department, Chulabhorn Hospital Chulabhorn Royal Academy Bangkok Thailand; ^6^ Department of Oral Health Ghent University Hospital Ghent Belgium; ^7^ Clinic of General, Special Care, and Geriatric Dentistry, Center for Dental Medicine University of Zurich Zurich Switzerland; ^8^ Center of Excellence in Genomics and Precision Dentistry, International Program in Geriatric Dentistry and Special Care, Faculty of Dentistry Chulalongkorn University Bangkok Thailand; ^9^ Division of Gerodontology and Removable Prosthodontics, University Clinics of Dental Medicine University of Geneva Geneva Switzerland

**Keywords:** geriatric dentistry, meta‐analysis, oral health, socioeconomic factors, systematic review

## Abstract

**Background:**

Oral diseases remain disproportionately prevalent among older adults. However, evidence on oral health inequalities among older adults remains dispersed across studies that have used different socioeconomic indicators and oral health measures and has not been synthesised.

**Objective:**

To synthesise the evidence on the association between socioeconomic factors and oral health among older adults aged 75 years and older.

**Methods:**

A systematic review and meta‐analysis was conducted following PRISMA guidelines. The Medline, Embase, and CENTRAL databases were searched. Studies reporting socioeconomic factors (education, income, occupation, area‐level deprivation, and multi‐aspect socioeconomic position) and oral health (dentition status, dental caries, periodontal disease, dry mouth, oral function, oral health behaviours, and oral health‐related quality of life (OHRQoL)) among older adults were included. Risk of bias was assessed using the Newcastle–Ottawa Scale.

**Results:**

Sixty‐eight studies were included. Meta‐analyses showed that socioeconomic disadvantage in older adults was associated with: (1) poor dentition status: fewer natural teeth, higher prevalence of edentulism, and lacking a functional dentition; (2) more teeth with decay; (3) irregular dental attendance; and (4) poorer OHRQoL. Similar patterns were generally observed for periodontal disease, dry mouth, and oral function, although no meta‐analysis could be performed due to limited evidence and heterogeneous oral health measures.

**Conclusion:**

Socioeconomic disadvantage was consistently associated with poor oral health in older adults. Associations were more pronounced for dentition status, reflecting the cumulative socioeconomic disadvantage over the life course. Socioeconomic factors should be considered to inform prevention, clinical decision‐making and oral healthcare planning for the ageing population.

**Trial Registration:**

PROSPERO Registration CRD420251231319

## 1 Introduction

Despite being largely preventable, oral diseases are highly prevalent, affecting over 3.7 billion people worldwide, including older adults [1]. As a result of ageing societies and changing oral epidemiology, older adults are now living longer and with heavily restored natural teeth and dental prostheses, thereby increasing the risk of oral conditions and complex treatment needs later in life [2]. Oral conditions considerably affect oral function, nutrition, and quality of life [3, 4]. Moreover, they are socially patterned, with persistent oral health inequalities documented across the life course [5, 6] and across different contexts and welfare states [7, 8], indicating the influence of social and structural health determinants.

Socioeconomic factors, usually represented by a range of interrelated constructs, notably education, income and occupation, represent a key determinant of oral health over the life‐course [6, 9]. Education influences values, preventive behaviours, and self‐care, and contributes to income through improved job prospects [10]. Income and occupation affect the affordability of dental care, a healthy diet and oral hygiene products [11, 12, 13]. Occupational conditions may affect oral health through psychosocial and environmental stressors [14]. This lifelong unequal exposure to resources, risks and opportunities can lead to profound inequalities in oral health, particularly among older adults.

While the influence of socioeconomic factors on oral health is well established, there is no appropriate synthesis of the strength and consistency of socioeconomic inequalities in oral health among older adults. Previous reviews have focused primarily on adults and on a single aspect of oral health, such as dental caries or periodontal disease [15, 16]. An earlier systematic review and meta‐analysis examined the influence of socioeconomic factors on multiple aspects of oral health, but not among older adults [17]. Another systematic review examined this, focusing mainly on institutionalised older adults, though not specifically on the population aged 75 years and over, and conducted a qualitative synthesis alone [18]. Moreover, oral health aspects that are particularly relevant for this age group, such as dry mouth and oral function, were not included. The relevant evidence is dispersed across studies that use different socioeconomic indicators and oral health measures, limiting generalisability and the ability to draw conclusions.

To address this knowledge gap, a comprehensive systematic review and meta‐analysis of the evidence on the association between socioeconomic factors and oral health among older adults was conducted. The aim was to synthesise evidence on the association between socioeconomic factors, captured by education, income, occupation and multi‐aspect measures, and oral health (dentition status, dental caries, periodontal disease, dry mouth, oral function, oral health behaviours, and oral health‐related quality of life) in older adults aged 75 years and over.

## 2 Methods

This review was conducted following the Preferred Reporting Items for Systematic Reviews and Meta‐Analyses (PRISMA) guidelines [19]. It was registered in the International Systematic Review Registry (PROSPERO; Registration number: CRD420251231319).

### 2.1 Search Strategy

The databases Medline (PubMed), Embase, and the Cochrane Central Register of Controlled Trials (CENTRAL) were searched for literature published from inception to 15th November 2025, in accordance with the eligibility criteria. The search strategy was devised in common for this paper and a related systematic review on the association between social support and oral health in the same population group and the detailed eligibility criteria and search terms used are presented in Table 1.

**TABLE 1:** Research question, inclusion and exclusion criteria and search terms.

Focus question	What is the association of socioeconomic factors with oral health in older adults?	
Criteria	Inclusion criteria	(1) Studies reporting on the impact of socioeconomic factors on oral health in older adults (≥ 75 years), or the study population had a mean age of 75 years (~75). (2) Socioeconomic context includes factors such as income level, education level, occupation, access to dental services, healthcare system structures and policy‐related influences. (3) Oral health includes clinical indicators (e.g., caries, periodontal disease, tooth loss), functional measures (chewing ability, pain), and behavioural factors (dental visits, oral hygiene habits). (4) Sample size ≥ 10 participants per study group
	Exclusion criteria	(1) Age < 75 years. (2) Case reports/series
Search terms	Population	#1 [MeSH]: Dental Care for Aged #2 [All fields]: Elder* OR Geriatric Population* OR Geriatric Patient* OR Ageing Population OR Aged 60 OR 60 year* OR Gerodont* OR Aged 75 OR 75 year* OR Aged 80 OR 80 year* OR Senior* OR Care‐depend* OR Older Adults OR Older People
	Intervention or exposure	#3 [MeSH]: Social support OR Social determinants of health OR Socioeconomic factors OR Health inequities OR Health services accessibility #4 [All fields]: Health inequity OR Oral health disparities OR Social isolation OR Community engagement OR Caregiver support OR Family support OR Income level OR Low‐income OR High‐income OR Economic status OR Education level OR Health literacy OR Access to dental care
	Outcome	#5 [MeSH]: Oral Health OR Tooth Loss OR Jaw, Edentulous OR Tooth demineralization OR Periodontal Diseases OR Dental pulp diseases OR Dentine sensitivity OR Quality of Life OR Xerostomia OR Mucositis OR Stomatitis OR Candidiasis, Oral #6 [All fields]: tooth loss OR edent* OR missing teeth OR Caries OR Carious OR Decay* OR DMF‐T OR DMFT OR demineralization OR periodont* OR Peri‐implant* OR Peri‐mucositis OR hypersensitivity OR Tooth sensitivity OR oral health‐related quality of life OR OHRQoL OR Oral Health Impact Profile OR OHIP OR Oral Impact on Daily Performances OR OIDP OR Self‐reported oral health OR Perceived oral health OR Xerostomia OR Mucositis OR Stomatitis OR Candidiasis OR Hyposalivation OR Dry mouth
Search Builder	(#1 OR #2) AND (#3 OR #4) AND (#5 OR #6)	

### 2.2 Study Selection and Data Extraction

The search results from the databases were imported into Rayyan, an online tool for managing systematic reviews (available at www.rayyan.ai). Duplicates were removed, and the two reviewers (SS and SP) independently screened the titles and abstracts of all articles for eligibility. Then, the full texts of potentially eligible studies were retrieved and reviewed. In cases where potentially eligible studies were published in languages other than English, translations were obtained using DeepL, a non‐commercial AI‐based online translation platform (https://www.deepl.com), to facilitate data extraction and interpretation. This happened for one article and the translated article was quantitative and straightforward to interpret. It was cross‐checked by two researchers (SP and SS) to ensure accuracy. Subsequently, any discrepancies were resolved through consensus among all authors. To maintain consistency in the screening process, inter‐investigator reliability was evaluated through Cohen's unweighted kappa (κ) [20].

The data were then extracted and summarised in a pre‐structured Excel sheet. To maximise the inclusion of available evidence, the extraction process was not limited to the main findings of each included study but also covered baseline data that were relevant to the research question. Studies were included if they reported data on individuals aged 75 years or older (even if the study also included younger adults), or if the study sample had a mean age of 75 years, with studies with a mean age of 74.5 years or higher rounded up to 75 years and therefore included. Information extracted included: author(s), publication year, title, sample country/countries, aim and objectives, study design, population studied (e.g., community‐dwelling, institutionalised, or mixed), age (mean or youngest), study duration (where available), sample size, socioeconomic factors, oral health measure(s) and the type of data available (e.g., cross‐sectional or cohort or both). The data extracted were independently cross‐checked by the two reviewers (SS and SP).

Socioeconomic factors were measured in the extracted manuscripts by a range of indicators such as income, occupation, education, and social class [9]. Across studies, these indicators were inconsistently reported, while some studies have also used measures that combine multiple socioeconomic factors, such as neighbourhood deprivation, which we refer to as multi‐aspect. In this review, socioeconomic factors are presented through two different ways: (1) socioeconomic position (SEP), which refers to income, occupation, and a multi‐aspect parameter, whereas (2) education was addressed separately. This was done to allow for separate meta‐analyses of the two most commonly reported relevant factors, that is, income and education, as a single socioeconomic parameter had to be selected to avoid double‐counting. For the meta‐analyses on the SEP, income was prioritised, and occupation was used when income was missing from the data. No studies reported a multi‐aspect SEP measure in combination with income or occupation. The oral health aspects identified in this review are reported using five domains: (1) dentition status, (2) oral conditions (dental caries, periodontal disease, dry mouth), (3) oral function, (4) oral health behaviours, and (5) oral health‐related quality of life (OHRQoL).

### 2.3 Quality Assessment

The risk of bias in the included studies was assessed based on the type of data included. This was independently evaluated by both reviewers (SS and SP) using the Newcastle–Ottawa Scale (NOS) for cohort studies [21] when the meta‐analysis used cohort data, and an adapted version of the NOS [22] when the meta‐analysis included cross‐sectional studies.

### 2.4 Data Synthesis

In addition to the analyses provided in the included studies, any relevant data from the descriptive analyses (e.g., sample sizes for those aged ≥ 75 years or for specific socioeconomic categories) that could be linked to provide derived estimates on the association between socioeconomic factors and oral health were extracted and analysed using unadjusted odds ratios (ORs) for categorical outcomes and mean differences (MDs) for continuous outcomes. Where feasible, meta‐analyses of evidence on the association between oral health and socioeconomic factors were performed by subgroup across SEP and education categories. These results were further stratified by population (e.g., institutionalised, community‐dwelling, or mixed) to provide greater granularity. Studies were excluded from the meta‐analyses if only adjusted estimates were available, if the available data were insufficient for calculating unadjusted effects, or if the included studies used convenience sampling. These studies, along with those reporting varied oral health measures, which could not be merged for meta‐analysis, were summarised narratively.

The risk of publication bias was evaluated using a funnel plot, which was generated when at least five studies were available. Funnel plots were also used for sensitivity analyses to identify studies that most strongly influenced the meta‐analysis in cases of moderate to high heterogeneity. For studies reporting results based on the same sample, only the most recent data were included in the meta‐analyses. All statistical analyses were conducted in RStudio (version 4.5.0) using the meta package.

## 3 Results

**FIGURE 1:**
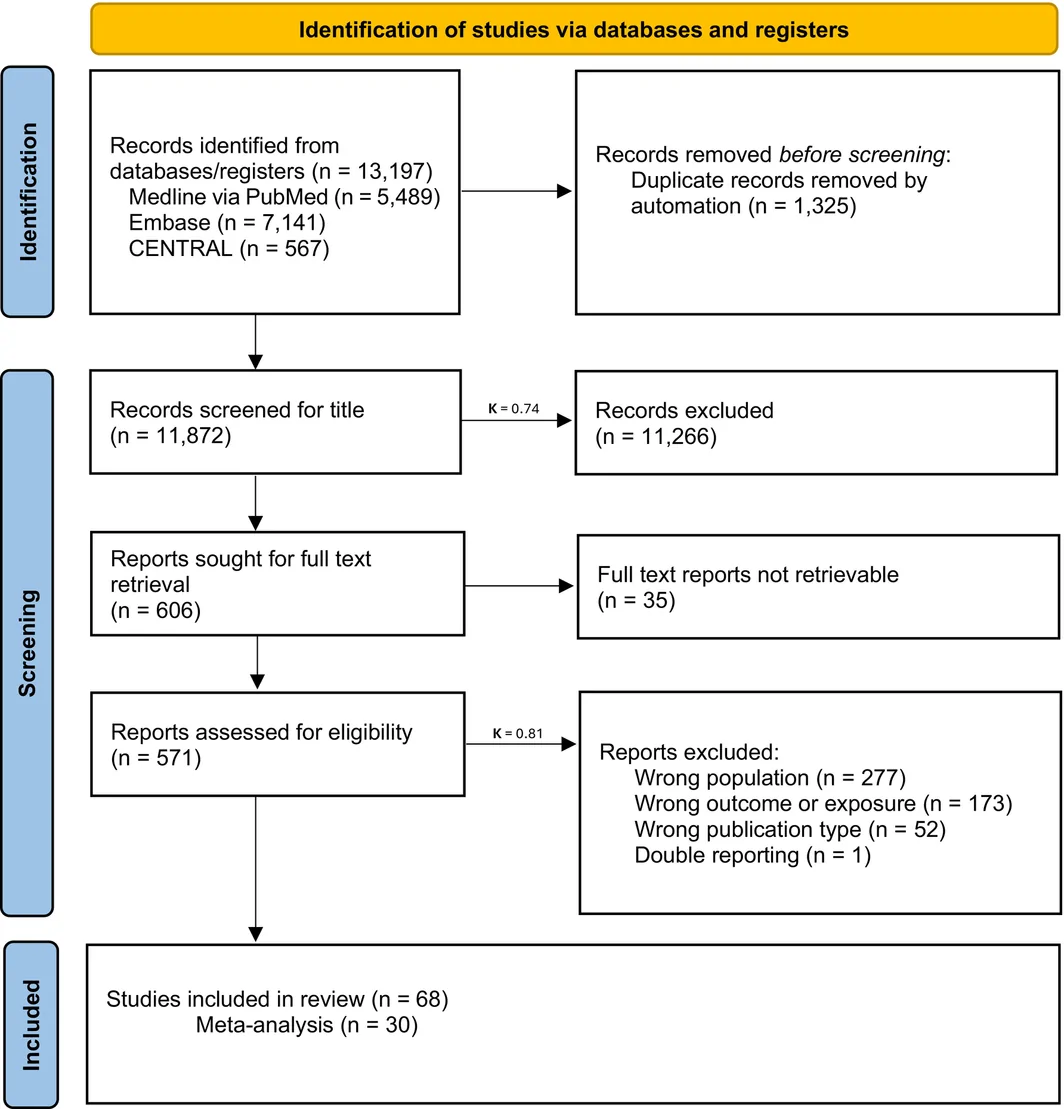
PRISMA 2020 flow diagram of study selection. [Colour figure can be viewed at wileyonlinelibrary.com]

The search yielded 13,197 records, of which 11,872 remained after removing duplicates in Rayyan. There was high consistency in the independent screening for the title and abstract, and for full text, with kappa values of 0.74 and 0.81, respectively (Figure 1). The excluded studies with reasons for exclusion are provided in Appendix S1.

### 3.1 Study Characteristics

A total of 68 studies were included in this review [23, 24, 25, 26, 27, 28, 29, 30, 31, 32, 33, 34, 35, 36, 37, 38, 39, 40, 41, 42, 43, 44, 45, 46, 47, 48, 49, 50, 51, 52, 53, 54, 55, 56, 57, 58, 59, 60, 61, 62, 63, 64, 65, 66, 67, 68, 69, 70, 71, 72, 73, 74, 75, 76, 77, 78, 79, 80, 81, 82, 83, 84, 85, 86, 87, 88, 89, 90]. The majority of extracted data used were from cross‐sectional studies [23, 24, 25, 26, 27, 28, 29, 30, 31, 32, 33, 34, 35, 36, 37, 38, 39, 40, 41, 42, 43, 44, 45, 46, 47, 48, 49, 50, 51, 52, 53, 54, 55, 56, 57, 58, 59, 60, 61, 62, 63, 64, 66, 67, 68, 69, 70, 71, 72, 73, 74, 75, 76, 77, 78, 79, 80, 81, 82, 83, 84, 85, 86, 87, 88, 89, 90], with only three studies providing cohort data [35, 65, 74]. One included study was in Japanese [85]. The study populations predominantly included community‐dwelling older adults, with a smaller number of studies conducted among institutionalised populations (*n* = 25) [29, 30, 33, 34, 38, 45, 46, 47, 49, 52, 58, 59, 61, 65, 67, 69, 72, 79, 81, 84, 86, 87, 88, 89, 90]. All included studies are described in Table 2.

**TABLE 2:** Included studies with the association of socioeconomic factors and oral health in older adults.

Authors, year	Country	Study design	Type of data	Population	Age mean (SD)	Sample size^a^	Socioeconomic factors	Oral health domains				
								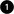	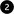	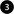	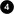	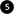
Aida et al. 2011 [23]	Japan	Longitudinal and cross‐sectional	Cross‐sectional	Community‐dwelling	74.9 (6.6)	21,736	Education	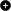				
Avlund et al. 2005 [24]	Sweden	Longitudinal and cross‐sectional	Cross‐sectional	Community‐dwelling	> or equal 80	157	SEP (occupation), Education	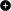	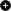		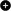	
Avlund et al., 2001 [25]	Sweden	Cohort	Cross‐sectional	Community‐dwelling	> or equal 75	411	SEP (income), Education	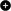		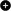	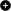	
Bakker et al. 2021 [26]	Netherlands	Longitudinal cohort	Cross‐sectional	Community‐dwelling	> or equal 75	168,122	SEP (multi‐aspect)	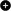				
Brothwell et al., 2008 [27]	Canada	Cross‐sectional	Cross‐sectional	Community‐dwelling	76.2 (7.1)	1751	SEP (income, occupation), Education				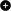	
Castrejón‐Pérez et al., 2017 [28]	Mexico	Longitudinal and cross‐sectional	Cross‐sectional	Community‐dwelling	79.2 (7.1)	647	Education					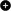
Cocco et al., 2018 [29]	Italy	Cross‐sectional	Cross‐sectional	Institutionalised	84.1 (9.7)	1654	SEP (multi‐aspect)	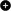				
Dai et al., 2023 [30]	China	Cohort and cross‐sectional	Cross‐sectional	Community‐dwelling and institutionalised	85.4 (10.5)	5403	Education	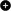				
Dye et al., 2019 [31]	US	Cross‐sectional	Cross‐sectional	Community‐dwelling	> or equal 75	11,476	SEP (income)	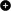				
Edman et al., 2016 [32]	Sweden	Cross‐sectional	Cross‐sectional	Community‐dwelling	equal 75, equal 85	864	SEP (income), Education		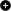			
Evren et al., 2011 [33]	Turkey	Cross‐sectional	Cross‐sectional	Institutionalised	76.5 (7.9)	269	SEP (income)				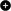	
Ferreira et al., 2008 [34]	Brazil	Cross‐sectional	Cross‐sectional	Institutionalised	79.0 (9.1)	335	SEP (income), Education	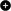				
Ganbavale et al., 2024 [35]	Britain	Cohort and cross‐sectional	Cohort and cross‐sectional	Community‐dwelling	78.7	2137	SEP (multi‐aspect)	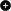	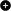			
Go et al., 2022 [36]	Korea	Retrospective cohort	Cross‐sectional	Community‐dwelling	> or equal 75	Not mentioned	SEP (income)	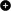				
Griffin et al., 2012 [37]	US	Cross‐sectional	Cross‐sectional	Community‐dwelling	> or equal 75	Not mentioned	SEP (income)	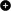	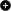			
Hassel et al., 2006 [38]	Germany	Cross‐sectional	Cross‐sectional	Institutionalised	83.3	158	Education					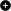
Hatta et al., 2018 [39]	Japan	Cohort	Cross‐sectional	Community‐dwelling	between 79 ‐ 81	463	SEP (income), Education	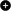				
Heegaard et al. [40]	Denmark	Longitudinal and cross‐sectional	Cross‐sectional	Community‐dwelling	75.5	782	SEP (income), Education	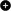				
Holm‐Pedersen et al., 2006 [41]	Sweden	Longitudinal and cross‐sectional	Cross‐sectional	Community‐dwelling	> or equal 80	101	SEP (occupation), Education		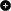			
Hsu et al., 2013 [42]	Taiwan	Cross‐sectional	Cross‐sectional	Community‐dwelling^b^	76.0 (0.4)	332	SEP (income), Education					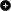
Hunt et al., 1985 [42]	US	Cross‐sectional	Cross‐sectional	Community‐dwelling	> or equal 75	3600	SEP (occupation), Education	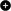				
Inomata et al., 2017 [44]	Japan	Longitudinal and cross‐sectional	Cross‐sectional	Community‐dwelling	between 79 ‐ 81	760	SEP (income), Education	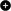				
Iosif et al., 2021 [45]	Romania	Cross sectional	Cross‐sectional	Institutionalised	81.5 (median)	50	Education					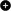
Islas‐Granillo et al., 2016 [46]	Mexico	Cross‐sectional	Cross‐sectional	Institutionalised	79.1 (9.8)	139	Education	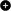				
Islas‐Granillo et al., 2014 [47]	Mexico	Cross‐sectional	Cross‐sectional	Institutionalised	79.1 (9.8)	139	Education		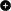			
Iwasaki et al.,2018 [48]	Japan	Cohort	Cross‐sectional	Community‐dwelling	75.0	322	SEP (income), Education	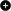				
Janssens et al. [49]	Belgium	Cross‐sectional	Cross‐sectional	Institutionalised	83.9 (8.5)	712	SEP (income)	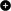				
Jensen et al., 2008 [50]	US	Cross‐sectional	Cross‐sectional	Community‐dwelling	79.1 (7.5)	641	SEP (income)					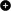
Kim et al. 2018 [51]	Korea	Cross‐sectional	Cross‐sectional	Community‐dwelling	82.7	241	Education	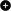		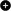		
Kim et al., 2009 [52]	Korea	Cross‐sectional	Cross‐sectional	Community‐dwelling and institutionalised	75.4 (7.7)	409	SEP (income), Education					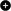
Komiyama et al., 2016 [53]	Japan	Cohort	Cross‐sectional	Community‐dwelling	75.2 (4.4)	834	Education	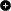			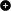	
Komulainen et al., 2012 [54]	Finland	Cross‐sectional	Cross‐sectional	Community‐dwelling	80.6 (3.6)	168	Education				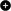	
Krustrup et al., 2008 [55]	Denmark	Longitudinal and cross‐sectional	Cross‐sectional	Community‐dwelling	equal 85	176	SEP (income, occupation), Education	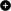	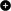			
Lee et al., 2022 [56]	Taiwan	Cross‐sectional	Cross‐sectional	Community‐dwelling^b^	74.7 (8.2)	202	SEP (income), Education					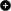
Liang et al., 2020 [57]	Taiwan	Cross‐sectional	Cross‐sectional	Community‐dwelling^b^	79.0	166	SEP (income), Education			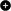		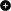
Ling et al., 2014 [58]	New Zealand	Case series and cross‐sectional	Cross‐sectional	Institutionalised^b^	82.6 (6.6)	179	SEP (multi‐aspect)	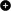	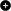			
Lundgren et al., 1995 [59]	Sweden	Cross‐sectional	Cross‐sectional	Community‐dwelling and institutionalised	equal 88	354	Education				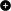	
Musacchio et al., 2007 [60]	Italy	Longitudinal and cross‐sectional	Cross‐sectional	Community‐dwelling	76.8 (8.7)	3054	SEP (income), Education	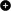				
Niesten et al., 2017 [61]	Netherlands	Cross‐sectional	Cross‐sectional	Institutionalised	85.4 (7.1)	126	SEP (multi‐aspect)				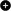	
Osterberg et al., 1995 [62]	Sweden, Denmark, Finland	Cross‐sectional	Cross‐sectional	Community‐dwelling	equal 75	945	SEP (income)	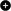				
Paulander et al., 2003 [63]	Sweden	Cross‐sectional	Cross‐sectional	Community‐dwelling	equal 75	238	Education	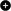	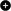			
Phipps et al., 2009 [64]	US	Longitudinal and cross‐sectional	Cross‐sectional	Community‐dwelling^b^	74.6	1210	Education	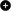	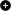			
Qi et al., 2023 [65]	China	Cohort and cross‐sectional	Cohort	Community‐dwelling and institutionalised	80.4 (9.7)	4268	SEP (income), Education	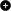				
Ramsay et al., 2015 [66]	Britain	Cross‐sectional	Cross‐sectional	Community‐dwelling	78.0	1612	SEP (occupation)	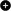	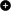			
Rapp et al., 2017 [67]	France	Cross‐sectional	Cross‐sectional	Community‐dwelling and institutionalised	82.5 (6.3)	1241	Education		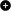			
Rise et al., 1982 [68]	Norway	Cross‐sectional	Cross‐sectional	Community‐dwelling	> or equal 75	530	Education				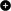	
Rocha‐Ortiz et al., 2023 [69]	Mexico	Cross‐sectional	Cross‐sectional	Institutionalised^b^	81.3 (9.0)	257	Education	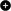				
Sato et al., 2016 [70]	Japan	Cohort and cross‐ sectional	Cross‐sectional	Community‐dwelling	equal 80	245	SEP (income), Education		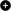			
Shimamura et al., 2022 [71]	Japan	Cross‐sectional	Cross‐sectional	Community‐dwelling	76.2 (7.4)	1616	SEP (income, occupation)	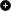				
Silva et al., 2014 [72]	Australia	Cross‐sectional	Cross‐sectional	Institutionalised	83.0	237	SEP (income)	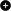	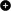			
Singhal et al., 2021 [73]	US	Cross‐sectional	Cross‐sectional	Community‐dwelling	> or equal 75	135,192	SEP (income)	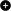				
Siukosaari et al., 2005 [74]	Finland	Cohort and cross‐sectional	Cohort and cross‐sectional	Community‐dwelling	> or equal 75	71	Education		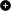			
Siukosaari et al., 2012 [75]	Finland	Cross‐sectional	Cross‐sectional	Community‐dwelling	> or equal 75	170	Education	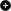	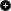			
Syrjälä et al.,2011 [76]	Finland	Cross‐sectional	Cross‐sectional	Community‐dwelling	79.8 (3.6)	154	Education		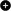			
Syrjälä et al., 2010 [77]	Finland	Cross‐sectional	Cross‐sectional	Community‐dwelling	> or equal 75	523	Education	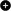				
Tennstedt et al., 1994 [78]	US	Cross‐sectional	Cross‐sectional	Community‐dwelling	77.5 (5.5)	633	Education				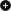	
Thorstensson et al., 2010 [79]	Sweden	Longitudinal and cross‐sectional	Cross‐sectional	Community‐dwelling and institutionalised	> or equal 80	357	SEP (income), Education	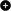				
Tsakos et al., 2009 [80]	UK	Cross‐sectional	Cross‐sectional	Community‐dwelling	74.7	1040	SEP (income), Education					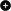
Uludamar et al., 2011 [81]	Turkey	Cross‐sectional	Cross‐sectional	Institutionalised	76.6 (9.2)	346	SEP (income)	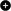	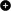		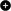	
Vu et al., 2022 [82]	Korea	Cross‐sectional	Cross‐sectional	Community‐dwelling	76	675	Education					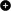
Wright et al., 2018 [83]	Australia	Longitudinal and cross‐sectional	Cross‐sectional	Community‐dwelling	> or equal 78	524	SEP (income)	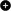	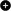			
Wu et al., 2018 [84]	China	Cross‐sectional	Cross‐sectional	Community‐dwelling and institutionalised	75.3 (6.7)	195	SEP (income, occupation), Education					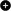
Yanagisawa et al., 2023 [85]	Japan	Cross‐sectional	Cross‐sectional	Community‐dwelling	75.1 (6.9)	739	SEP (income), Education	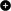				
Yang et al., 2022 [86]	China	Cohort and cross‐sectional	Cross‐sectional	Community‐dwelling and institutionalised	> or equal 80	5513	SEP (income), Education	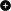				
Zini et al., 2008 [87]	Israel	Cross‐sectional	Cross‐sectional	Community‐dwelling and institutionalized^b^	75.7 (8.1)	344	SEP (income), Education					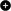
Zini et al., 2011 [88]	Israel	Case control and cross‐sectional	Cross‐sectional	Community‐dwelling and institutionalised^b^	75.7 (8.1)	344	SEP (income), Education				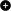	
Zuluaga et al., 2012 [89]	Spain	Cross‐sectional	Cross‐sectional	Institutionalised	82.9 (7.6)	295	Education					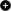
Chan et al., 2025 [90]	China	Cross‐sectional	Cross‐sectional	Community‐dwelling and institutionalised	82.6 (11.2)	7796	SEP (income, occupation), Education	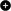				

*Note:* 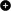, Oral health domains reported in the study; 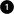, Dentition status; 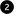, Oral conditions: dental caries, periodontal disease, and dry mouth; 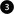, Oral function; 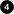, Oral health behaviours; 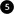, Oral Health‐Related Quality of Life; SEP: socioeconomic position.

^a^ Sample size reported based on the extracted data in this review.

^b^ Employed convenience samples.

### 3.2 Risk of Bias and Quality Assessment

The risk of bias and quality of the included cross‐sectional and cohort studies were assessed as ranging from moderate to low. Eight studies were considered to have low population representativeness due to employing convenience sampling [42, 56, 57, 58, 64, 69, 87, 88]. Detailed assessments are provided in Appendix S2 (Tables 1–3).

### 3.3 Synthesis of Results

The overall information on the associations between socioeconomic factors and oral health is presented in Appendix S3 (Table 1). Twelve meta‐analyses were conducted, of which four were further examined using funnel plots and demonstrated acceptable levels of publication bias. These included the associations between: (1) SEP and edentulism prevalence, (2) SEP and prevalence of lacking functional dentition (i.e., having fewer than 20 natural teeth), (3) education and edentulism prevalence, and (4) education and prevalence of lacking functional dentition (Appendix S4: Figures S20–S23).

#### 3.3.1 Dentition Status

This review identified key indicators of dentition status, including the number of missing teeth, edentulism prevalence, and prevalence of lacking functional dentition (*n* = 40) [23, 24, 25, 26, 29, 30, 31, 34, 35, 36, 37, 39, 40, 43, 44, 46, 48, 49, 51, 53, 55, 58, 60, 62, 63, 64, 65, 66, 69, 71, 72, 73, 75, 77, 79, 81, 83, 85, 86, 90].

**FIGURE 2:**
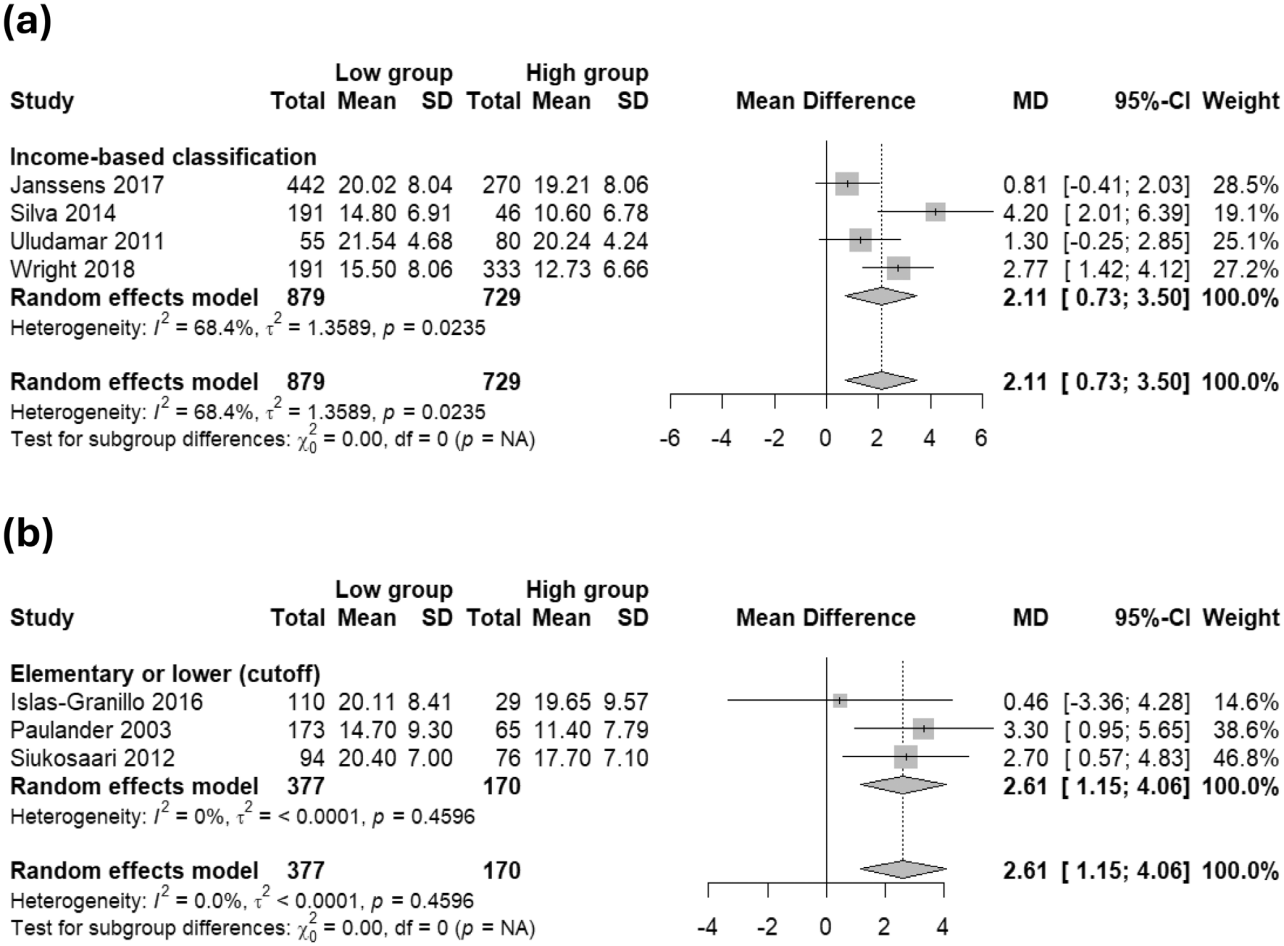
Forest plot showing the mean difference in the number of missing teeth. (a) Socioeconomic position; (b) Education level.

The meta‐analysis of SEP (Figure 2a), which used income‐based [49, 72, 81, 83] parameters, showed that older adults from low SEP had, on average, two fewer teeth than those from higher SEP (MD 2.11; 95% CI: 0.73, 3.50), with moderate heterogeneity (I^2^ = 68.4%). Population‐stratified analysis showed associations in both the community‐dwelling [83] (MD 1.93; 95% CI: 0.02, 3.84) and institutionalised [49, 72, 81] (MD 2.77; 95% CI: 1.42, 4.12) populations (Appendix S4: Figure S1). Similarly, meta‐analyses on education and missing teeth [46, 63, 75] documented that lower education levels were associated with a greater number of missing teeth (MD 2.61; 95% CI: 1.15, 4.06) than higher education levels, with no heterogeneity (Figure 2b). Population‐stratified analysis showed an association in community‐dwelling populations [63, 75] (MD 2.97; 95% CI: 1.39, 4.55) (Appendix S4: Figure S2). This association was also supported by three adjusted analyses [55, 65, 69], one each in a community population [55], institutionalised population [69], and a mixed sample comprising community‐dwelling and institutionalised older adults [65].

**FIGURE 3:**
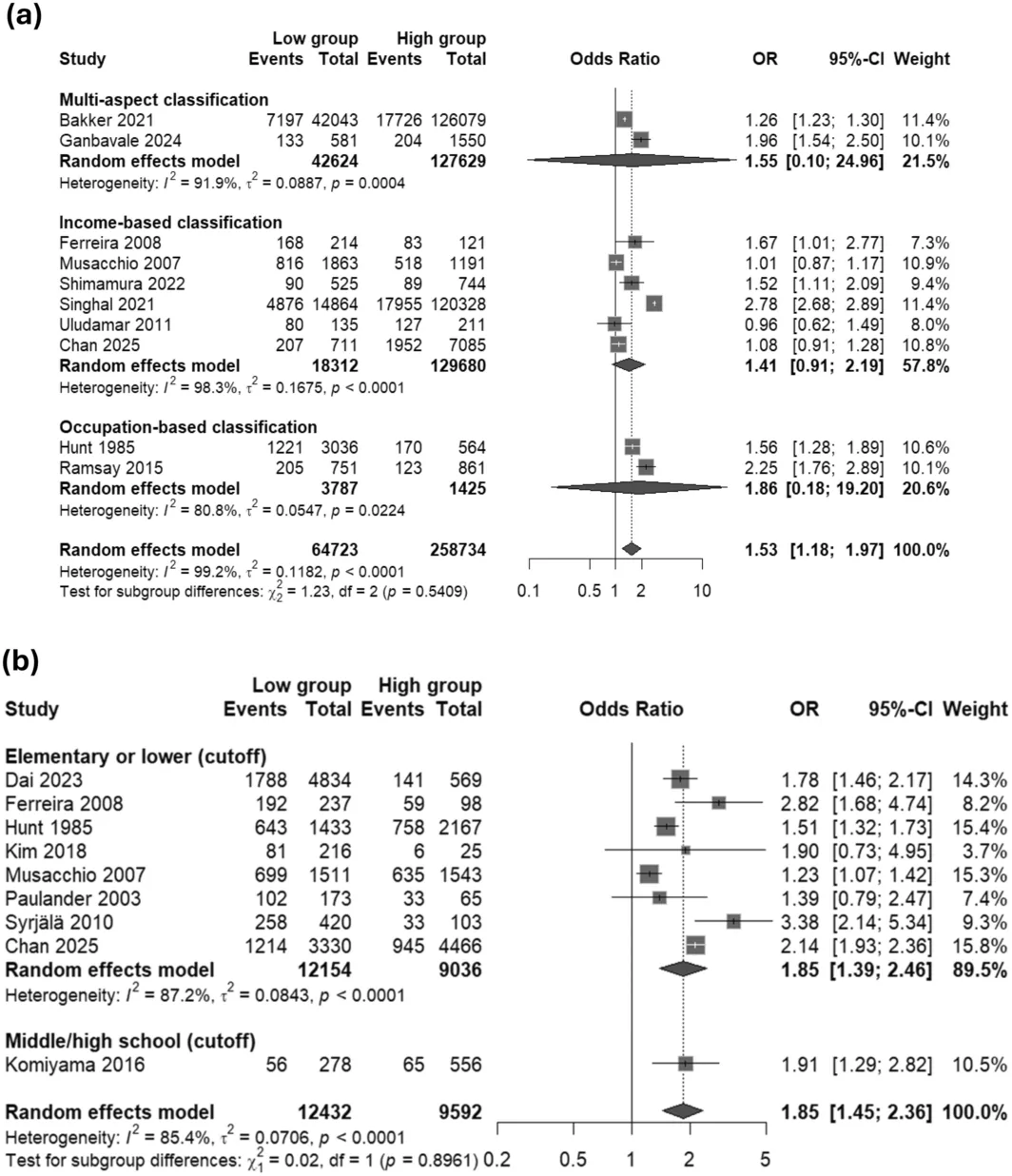
Forest plot showing the odds ratio in the prevalence of edentulism. (a) Socioeconomic position; (b) Education level.

**FIGURE 4:**
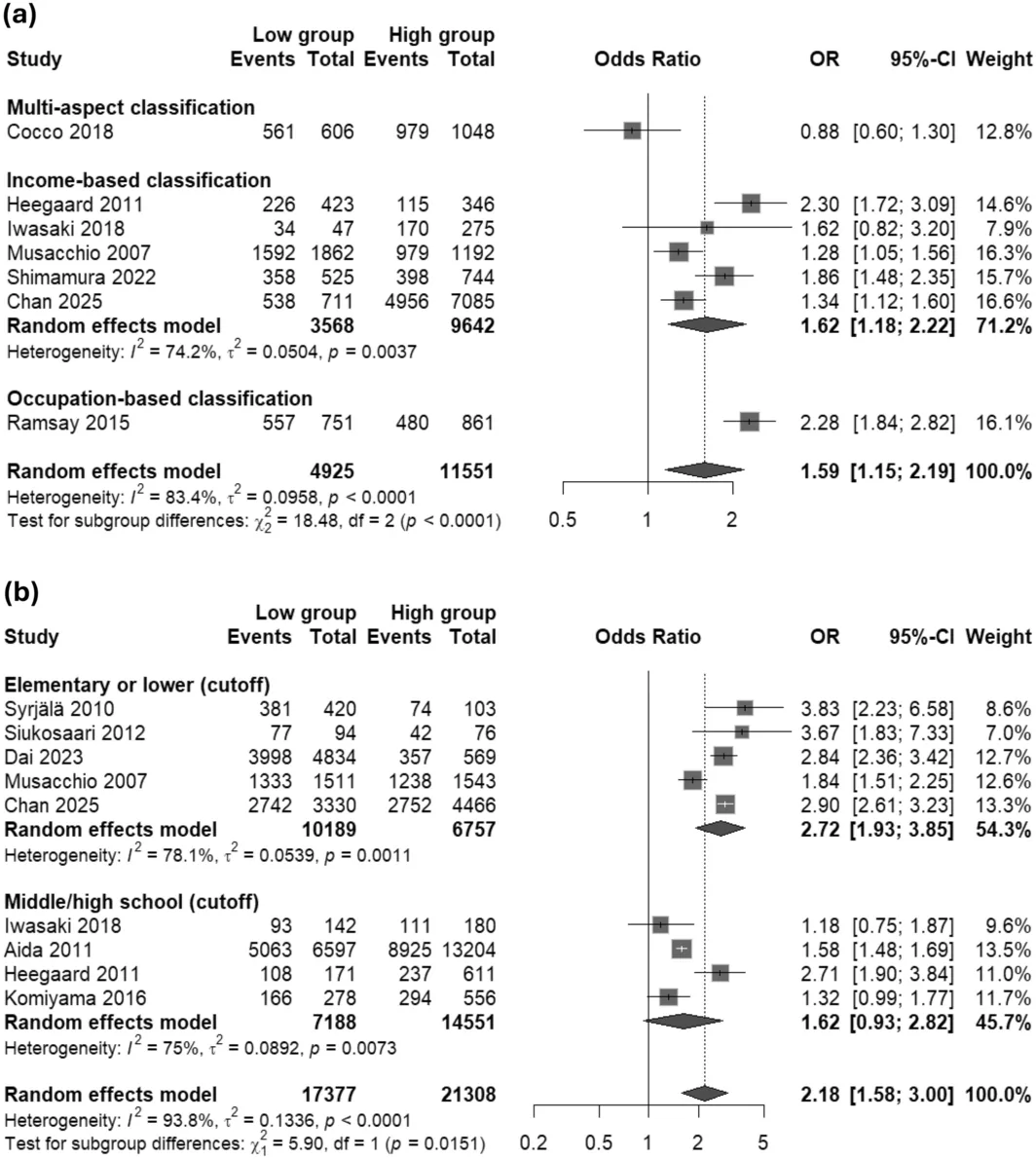
Forest plot showing the odds ratio in the prevalence of lacking a functional dentition. (a) Socioeconomic position; (b) Education level.

In the meta‐analyses of edentulism (*n* = 10) [26, 34, 35, 43, 60, 66, 71, 73, 81, 90] and lacking a functional dentition (*n* = 7) [29, 40, 48, 60, 66, 71, 90], older adults in lower SEP were more likely to experience edentulism (OR 1.53; 95% CI: 1.18, 1.97) and lack functional dentition (OR 1.59; 95% CI: 1.15, 2.19) than those in higher SEP, with both meta‐analyses showing high heterogeneity (Figures 3a and 4a). Population‐stratified analysis showed that lower SEP was associated with edentulism in community‐dwelling population samples [26, 35, 43, 60, 66, 71, 73] (OR 1.67; 95% CI: 1.21, 2.32) with high heterogeneity (I^2^ = 99.4%), whereas the evidence showed no association in institutionalised populations [34, 81] (OR 1.25; 95% CI: 0.04, 41.71) (Appendix S4: Figure S3). Similarly, associations were consistent across community‐dwelling populations [40, 48, 60, 66, 71] for lacking functional dentition (OR 1.84; 95% CI: 1.33, 2.54) (Appendix S4: Figure S4). Sensitivity analysis excluding the two outlier studies on the meta‐analysis on edentulism [26, 73] reduced heterogeneity from I^2^ = 99.2% to 86.6% without much altering the pooled estimate (Appendix S4: Figure S5). Sensitivity analysis for the prevalence of lacking functional dentition reduced heterogeneity from 83.4% to 74.2% while maintaining a comparable pooled estimate (Appendix S4: Figure S6).

Moreover, the meta‐analyses on education level documented similar associations, with low education being associated with a higher prevalence of edentulism (*n* = 9) [30, 34, 43, 51, 53, 60, 63, 77, 90] (OR 1.85; 95% CI: 1.45, 2.36) (Figure 3b) and lacking functional dentition (*n* = 9) [23, 30, 40, 48, 53, 60, 75, 77, 90] (OR 2.18; 95% CI: 1.58, 3.00) (Figure 4b), compared with higher education levels. Population‐stratified analyses by education showed associations in both community‐dwelling [43, 51, 53, 60, 63, 77] (OR 1.71; 95% CI: 1.17, 2.51) and institutionalised [34] (OR 2.82; 95% CI: 1.68, 4.74) populations for edentulism (Appendix S4: Figure S7). Low education was also associated with lacking functional dentition in community‐dwelling populations [23, 40, 48, 53, 60, 75, 77] (OR 1.98; 95% CI: 1.31, 3.00) and studies covering community‐dwelling and institutionalised populations [30, 90] (OR 2.89; 95% CI: 2.55, 3.27), with no study reporting exclusively from institutionalised settings (Appendix S4: Figure S8). Sensitivity analyses further reduced heterogeneity from I^2^ = 85.4% to 30.2% and from I^2^ = 93.8% to 83.5%, respectively, while preserving similar pooled estimates (Appendix S4: Figures S9 and S10). The remaining substantial heterogeneity in the prevalence of edentulism and lacking functional dentition across studies on SEP and education corresponded to variation observed in individual‐adjusted analyses, which reported both associations [25, 35, 62, 79] and no associations [23, 24, 60, 86] across community‐dwelling [23, 24, 25, 35, 60, 62] and mixed [79, 86] populations.

#### 3.3.2 Oral Conditions: Dental Caries, Periodontal Disease and Dry Mouth

For dental caries, meta‐analyses by SEP were conducted using the number of decayed teeth (*n* = 3) [72, 81, 83]. Older adults in low SEP had a slightly higher number of decayed teeth than those in higher SEP (MD 0.45; 95% CI: 0.15, 0.75), with no heterogeneity (Appendix S4: Figure S11). Population‐stratified analysis showed an association in a community‐dwelling population [83] (MD 0.52; 95% CI: 0.18, 0.86) (Appendix S4: Figure S12). This finding was consistent with a study that showed an association between SEP and the number of carious teeth in adjusted analyses [24]. Since the number of studies with education level was limited (*n* = 5) and the reported measures were heterogeneous, a meta‐analysis could not be performed for these studies [24, 32, 55, 63, 74]. Two of these studies [32, 63] reported associations in unadjusted analyses, while all three studies that performed adjusted analyses showed no associations [24, 55, 74].

For periodontal disease, 12 included studies [35, 37, 41, 63, 64, 66, 67, 70, 74, 75, 76, 83] reported associations with socioeconomic factors; however, meta‐analysis was not possible. Among studies that conducted adjusted analyses, one study, using a multi‐aspect SEP, demonstrated an association [35], whereas another study, using occupation‐based classification and education level, reported non‐significant results [41].

Lastly, two studies were included for the dry mouth [35, 47]. Of these, only one study used adjusted analysis and reported a higher prevalence of dry mouth (assessed through the Xerostomia Inventory Scale) among community‐dwelling older adults in more deprived neighbourhoods, in cross‐sectional data, but not in 8‐year cohort data [35].

#### 3.3.3 Oral Function

This review identified three studies [25, 51, 57] that examined the association between socioeconomic factors and oral function. A meta‐analysis could not be conducted due to the limited number of studies and the heterogeneity of measures. Adjusted analyses showed that community‐dwelling older adults with a sufficient but not affluent economic status and less than 3 years of education had higher odds of poor oral function [57].

#### 3.3.4 Oral Health Behaviours

Thirteen studies [24, 25, 27, 33, 53, 54, 59, 61, 68, 73, 78, 81, 88] examined the association between socioeconomic factors and oral health behaviours, primarily focusing on dental care attendance among older adults. Meta‐analysis with moderate heterogeneity (I^2^ = 58.8%) showed that older adults in low SEP were 2.42 (95% CI: 1.60, 3.66) times more likely to report irregular use of dental services compared with those in high SEP across both occupation [61] and income parameters [33, 73, 81] (Appendix S4: Figure S13). Population‐stratified analyses showed that associations were stronger in community‐dwelling [73] (OR 3.02; 95% CI: 2.92, 3.13) than in institutionalised populations [33, 61, 81] (OR 1.96; 95% CI: 1.40, 2.75) (Appendix S4: Figure S14). Adjusted analyses [24, 25, 27, 61, 88] reported the same direction of this association, with studies showing associations across community‐dwelling [25, 27] and mixed populations [88].

A meta‐analysis of two community‐dwelling population studies [53, 68] found no association between education level and dental attendance (OR: 3.05; 95% CI: 0.00, 6745.81) (Appendix S4: Figure S15). Several studies that conducted adjusted analyses reported a positive association between higher education level and more frequent dental visits [25, 27, 59, 78], while one reported no association [24].

Additionally, three studies [33, 61, 81] from institutionalised settings assessed the association between SEP and toothbrushing frequency. The pooled meta‐analysis yielded no association (OR 1.87; 95% CI: 0.44, 7.93) with moderate heterogeneity (I^2^ = 61.2%) (Appendix S4: Figure S16).

#### 3.3.5 OHRQoL

This review included 13 studies that examined the association between socioeconomic factors and OHRQoL [28, 38, 42, 45, 50, 52, 56, 57, 80, 82, 84, 87, 89], using two instruments: the Oral Health Impact Profile‐14 (OHIP‐14) (*n* = 8) [28, 38, 45, 50, 52, 56, 82, 87] and the Geriatric Oral Health Assessment Index (GOHAI) (*n* = 5) [42, 57, 80, 84, 89]. The meta‐analysis (*n* = 2) [52, 80] using a community‐dwelling sample [80] and a mixed population sample [52] showed that older adults with low income were associated with poorer OHRQoL (standardised mean difference: SMD 0.17; 95% CI: 0.07, 0.28) than those with higher SEP, with no heterogeneity (Appendix S4: Figure S17). Two individual studies also reported associations between low income and poor OHRQoL in adjusted analyses [52, 57], but others found no significant results [56, 80, 84, 87].

Furthermore, the meta‐analysis (*n* = 3) [28, 52, 80] indicated that older adults with low education levels reported a marginally poorer OHRQoL (SMD 0.21; 95% CI: 0.11, 0.30) than those with high education levels, with almost no heterogeneity (I^2^ = 0.4%) (Appendix S4: S18). Population‐stratified analysis revealed associations for community‐dwelling [28, 80] (SMD 0.23; 95% CI: 0.13, 0.34) and mixed population [52] samples (SMD 0.08; 95% CI: 0.13, 0.29) (Appendix S4: Figure S19). Findings from individual studies that reported adjusted analyses indicated mostly no associations across community‐dwelling [28, 56, 57, 82], institutionalised [89], and mixed [52, 84] populations, although three studies showed that low education was associated with poor OHRQoL across different populations [38, 80, 87]. In contrast, one study from Taiwan reported higher education to be associated with poorer OHRQoL [42].

## 4 Discussion

This systematic review and meta‐analysis provides comprehensive evidence that socioeconomic factors were consistently associated with poorer oral health and behaviours in older adults aged 75 years and over. More specifically, meta‐analyses documented that socioeconomic disadvantage was associated with poor dentition status (fewer teeth, higher prevalence of edentulism, and higher prevalence of lacking a functional dentition), higher numbers of decayed teeth, irregular dental attendance, and generally with poor OHRQoL. Overall, associations were stronger in meta‐analyses for dentition status than in any other meta‐analyses. Similar patterns were generally observed for periodontal disease, oral function, and dry mouth, although no meta‐analysis could be performed due to the limited available evidence and varied outcome measures.

To our knowledge, this is the most comprehensive systematic synthesis of evidence, including meta‐analysis, on the association of socioeconomic factors with several aspects of oral health in older adults (≥ 75). We have conducted robust sensitivity analyses, where appropriate, that supported the consistency of our findings and have used meta‐analysis cautiously, refraining from forcing synthesis when evidence was limited or outcomes were heterogeneous. Meta‐analysis was performed using unadjusted estimates, as few studies reported adjusted analyses. Findings should therefore be interpreted cautiously, given that the study associations are prone to confounding in general and in this population in particular, due to existing multi morbidities. To support interpretation, adjusted analyses were presented narratively alongside the meta‐analysis. As relevant cohort studies were scarce in this population, the included studies were limited by their predominantly cross‐sectional design; therefore, not allowing causal inferences. Considering also that participants were aged 75 years and over, the findings may also be affected by survival bias, with healthy individuals from lower socioeconomic groups more likely to survive than their unhealthy counterparts in the same socioeconomic category, which may potentially attenuate the associations. Another limitation is the possibility of reverse causality, which is difficult to disentangle in cross‐sectional data, thereby limiting interpretation.

The use of different sampling frames across the different studies may have affected the generalisability of the findings. Heterogeneity was high across several meta‐analyses, and this likely reflects variation in population contexts. To address this, we stratified analyses by studies employing community‐dwelling samples, those with institutionalised samples, and those using both community‐dwelling and institutionalised samples. This has helped highlight the potential role of the nature and context of the different samples used. Some unexplained variability remained in the population‐stratified analyses, potentially due to differences in measures, further limiting the generalisability of the pooled estimates. It is also worth noting the limited number of studies among institutionalised older adults. Moreover, the included studies were primarily conducted in the Global North, with very few undertaken in the Global South within this population group. Overall, the findings should be interpreted carefully, as the studies used a wide range of measurement instruments and outcomes, each with varying definitions.

Socioeconomic factors showed the strongest associations with dentition status among the several aspects of oral health studied here. Low SEP was associated with poor dentition status, including fewer teeth, higher edentulism prevalence, and a greater likelihood of functional dentition loss. Our findings are consistent with the literature showing that socioeconomic disadvantage is associated with poor dentition status [5, 91, 92, 93, 94, 95, 96, 97]. The findings emphasise that socioeconomic factors have a crucial role in shaping oral health, also in older adulthood. It is possible that this could be through biological, material, behavioural, and psychosocial mechanisms, potentially due to cumulative exposure to risk factors across the life course, which is predominantly seen in tooth loss, as it is the final outcome of oral diseases. Other studies have highlighted the influence of accumulated risk factors over the life course on dentition status [98, 99].

Socioeconomic factors were generally associated with dental caries, periodontal disease, and dry mouth, although evidence of association was inconsistent for periodontal disease and limited for dry mouth. Low SEP was associated with more decayed teeth in this meta‐analysis, and with periodontal disease and dry mouth in adjusted analyses [35]. It implies that SEP can adversely affect these oral conditions. Previous research in younger population groups showed that socioeconomic disadvantage was associated with a higher likelihood and greater severity of both dental caries and periodontal diseases [15, 16, 94, 100], highlighting the role of socioeconomic factors in these conditions. However, older adults aged 75 years or older retain fewer natural teeth, particularly those in lower SEP, and this may also be associated with a lower likelihood of oral diseases, which could partially explain the variable associations observed in this review. For dry mouth, the evidence was scarce, with only two studies and therefore should be interpreted cautiously.

Low education and non‐affluent economic status were generally associated in adjusted analyses with poor oral function [25, 51, 57], though this finding was based on only three studies and no meta‐analysis was conducted due to the limited available evidence. This association may be indirect, through poor dentition status. For example, chewing problems were higher among older edentulous participants [101] and chewing ability has been shown to be associated with childhood adversity [102]. Moreover, socioeconomic factors also influence the ability to seek treatment and the type of treatment, thereby potentially affecting oral function [103, 104, 105].

Socioeconomic factors were also associated with oral health behaviours, although education‐based differences in oral health behaviours were inconclusive. Low SEP was associated with irregular dental attendance [33, 61, 73, 81, 88]. This implies that low SEP may act as a barrier for dental attendance and thereby has the potential to influence other aspects of oral health, such as dentition status, dental caries, periodontal disease, and oral function. Studies have documented the generally modest role of behavioural pathways in explaining socioeconomic inequalities in oral health [7, 8, 95, 106, 107, 108, 109, 110]. Some qualitative studies highlighted cost as a barrier, which is related to socioeconomic factors [103, 104, 105, 111, 112]. Finally, poor SEP was associated with poor OHRQoL across different instruments [42, 52, 80]. OHRQoL captures the combined consequences of poor oral health on the quality of life. These findings are consistent with the broader literature on socioeconomic inequalities in OHRQoL [7, 8, 108, 109, 110].

The findings from this review highlight the relevance of socioeconomic factors for the oral health of older adults aged 75 years and older. At the population level, socioeconomic factors should be considered in health services planning, particularly in resource allocation and the development of suitable financial mechanisms to promote equity. At the clinical level, they can be considered in treatment planning and determining recall intervals, thereby enabling patient‐centred care. Future research could develop, test, and validate multi‐dimensional risk indices for older adults that integrate socioeconomic factors.

## 5 Conclusion

This systematic review and meta‐analysis showed that socioeconomic disadvantage was consistently associated with worse oral health and poorer behaviours among adults aged 75 years and older. Associations were more pronounced for dentition status, with people in socioeconomic disadvantage having a higher prevalence of edentulism, fewer teeth, and a higher prevalence of not having a functional dentition than their more affluent counterparts. Socioeconomic factors should be considered to inform prevention, clinical decision‐making, and oral healthcare planning for the ageing population.

## Author Contributions

F.M., G.T., B.J. and M.S. conceived the initial study idea. S.S.S. and S.P. conducted the research study together and were supported by F.M., G.T., B.J. and M.S. S.S.S. and S.P. prepared the first draft, and all authors were involved in reading, critically revising, editing and approving the final manuscript.

## Funding

The authors have nothing to report.

## Ethics Statement

The authors have nothing to report.

## Conflicts of Interest

The authors declare no conflicts of interest.

## Supporting information


**Appendix S1.** List of excluded studies with reasons for exclusion.

**Appendix S2.** Risk of bias and quality assessment of included studies.

**Appendix S3.** Summary of findings on the association of socioeconomic factors with oral health.

**Appendix S4.** Supplementary forest and funnel plots of meta‐analyses.

## Data Availability

The data analysed in this study are derived from previously published studies included in the systematic review. All data supporting the findings of this study are available within the article and its supporting information. No new primary data were generated for this study.
