# Supplementary material for: Association of Socioeconomic Factors With Oral Health in Older Adults: A Systematic Review and Meta‐Analysis
Source: Gerodontology. 2026 May 15;43(3):456–74. doi: 10.1111/ger.70079 (PMC13498931; doi:10.1111/ger.70079)
Supplement: Supplementary file 1 [file GER-43-456-s001.docx]

Appendix S1. List of excluded studies with reasons for exclusion.

| Reason | Author | Title |
| --- | --- | --- |
| wrong outcome or exposure | Wyatt (2009) | A 5-year follow-up of older adults residing in long-term care facilities: utilisation of a comprehensive dental programme |
| wrong population | Beck (1997) | A 5-year study of attachment loss in community-dwelling older adults: incidence density. |
| wrong population | Gopalakrishnan (2019) | Access and barriers to oral health care for dependent elderly people living in rest homes. |
| wrong population | Folorunsho (2025) | Access to and Utilization of Dental Care Services by Older Adults in Nigeria: Barriers and Facilitators. |
| wrong outcome or exposure | Sória (2019) | Access to and use of oral health services among the elderly in Pelotas, Rio Grande do Sul State, Brazil |
| wrong population | Crocombe (2022) | Access to dental care barriers and poor clinical oral health in Australian regional populations |
| wrong population | Ramirez-Ortega (2023) | Access to Dental Services among Hypertensive Elderly in Peru: Exploring Patterns and Implications |
| wrong population | Mariño (2001) | Acculturation and dental health among Vietnamese living in Melbourne, Australia |
| wrong population | Northridge (2015) | A community-based oral public health approach to promote health equity. |
| wrong population | Mascarenhas (1999) | A comparison of oral health in elderly populations seeking and not seeking dental care. |
| wrong population | Prasanna (2012) | A cross-sectional study on the relationship between behavioral variables and economic status in periodontal disease among elderly individuals. |
| wrong publication type | Lowenstein (2025) | Addressing disparities in oral health access and outcomes for aging adults in the United States. |
| wrong population | Tomar (1998) | Adult dental visits in California: successes and challenges |
| wrong population | Zhang (2023) | Adverse Childhood Experiences and Oral Health Conditions Among Middle-aged and Older Chinese Adults: Exploring the Moderating Roles of Education and Gender |
| wrong outcome or exposure | Limo (2024) | Age and sex differences in the association of dental visits with inadequate oral health and multimorbidity: Findings from the Canadian Longitudinal Study on Aging (CLSA) |
| wrong population | Li (2011) | Age, period, and cohort analysis of regular dental care behavior and edentulism: a marginal approach |
| wrong population | Rademakers (2008) | Aging and oral health care in The Netherlands. An explorative study |
| wrong outcome or exposure | Lee (2019) | A life course approach to total tooth loss: Testing the sensitive period, accumulation, and social mobility models in the Health and Retirement Study. |
| wrong outcome or exposure | Mendes (2012) | Analysis of socio-demographic and systemic health factors and the normative conditions of oral health care in a population of the Brazilian elderly. |
| wrong outcome or exposure | Miremadi (2017) | A pilot assessment tool of the need for oral health care and cost prediction in institutionalized elderly people |
| wrong outcome or exposure | Ikebe (2004) | Application of short-form oral health impact profile on elderly Japanese. |
| wrong population | Khalaila (2024) | A prospective association between social isolation and cognitive performance among older adults in Europe: the role of loneliness and poor oral health. |
| wrong population | Chen (1986) | A sociodemographic analysis of preventive dental behavior among white American families |
| wrong population | Kim (2021) | Assessment of age-related changes on masticatory function in a population with normal dentition |
| wrong outcome or exposure | Meyer-Hofmann (2025) | Assessment of Oral Health Literacy and Influencing Factors: Retrospective Analysis of Patients Treated by Dental Students. |
| wrong outcome or exposure | León (2019) | Assessment of oral health-related quality of life as a function of non-invasive treatment with high-fluoride toothpastes for root caries lesions in community-dwelling elderly |
| wrong population | Nawaz (2022) | Association among four aspects of Oral Wellness Standard of Living and Wellness Quality of Life Concepts in the Dental Patient Sample |
| wrong outcome or exposure | Xin (2024) | Association and mediators between the impact of oral health-related quality of life and depression. |
| wrong outcome or exposure | Da Silva (2021) | Association between cognitive health and masticatory conditions: a descriptive study of the national database of the universal healthcare system in Japan. |
| wrong population | Tan (2022) | Association between dentition and frailty and cognitive function in community-dwelling older adults. |
| wrong population | Kim (2023) | Association between depressive mood and chronic periodontitis among senior residents using the National Health Insurance Service-Senior Cohort Database. |
| wrong population | Allen (2005) | Association between diet, social resources and oral health related quality of life in edentulous patients |
| wrong outcome or exposure | Tashiro (2017) | Association between income inequality and dental status in Japanese older adults: Analysis of data from JAGES2013 |
| wrong outcome or exposure | Saito (2021) | Association Between Oral Health and the Medical Costs of Dementia: A Longitudinal Study of Older Japanese |
| wrong publication type | Borg-Bartolo (2025) | Association between oral health-related quality of life and structural determinants of health among elderly populations. a systematic review and meta-analysis. |
| wrong outcome or exposure | Diaz-Toro (2022) | Association between Poor Oral Health and Frailty in Middle-Aged and Older Individuals: A Cross-Sectional National Study |
| cannot retrieve full text | Hao (2022) | Association between self-reported gingival bleeding and prevalent hypertension among adults in a cross-sectional study in Zhejiang province |
| wrong outcome or exposure | Wang (2023) | Association between self-reported oral health and life satisfaction among China's migrant elderly following children: The mediating effect of social support. |
| wrong population | Alqaderi (2020) | Association between sleep and severe periodontitis in a nationally representative adult US population |
| wrong outcome or exposure | Olofsson (2018) | Association between socioeconomic and health factors and edentulism in people aged 65 and older - a population-based survey. |
| wrong population | Oliveira (2018) | Association between socioeconomic factors, behavioral, general health and oral mucosa status in elderly. |
| wrong population | Park (2016) | Association between socioeconomic status and oral health behaviors: The 2008-2010 Korea national health and nutrition examination survey |
| wrong outcome or exposure | Yamamoto (2014) | Association between the longest job and oral health: Japan Gerontological Evaluation Study project cross-sectional study. |
| wrong outcome or exposure | Castrejón-Pérez (2012) | Association between the Use of Complete Dentures and Frailty in Edentulous Mexican Elders. |
| wrong population | Luo (2015) | Association between tooth loss and cognitive function among 3063 Chinese older adults: A community-based study |
| wrong outcome or exposure | Xu (2023) | Association between tooth loss and frailty among Chinese older adults: the mediating role of dietary diversity. |
| wrong outcome or exposure | Iwasaki (2017) | Association between tooth loss and medical costs related to stroke in healthy older adults aged over 75 years in Japan |
| wrong outcome or exposure | Gao (2016) | Association of cognitive function with tooth loss and mitochondrial variation in adult subjects: a community-based study in Beijing, China |
| wrong population | Amoah (2022) | Association of Health Literacy and Socioeconomic Status with Oral Health Among Older Adults in Ghana: A Moderation Analysis of Social Capital. |
| wrong publication type | Ramsay (2017) | Association of objective and perceived neighbourhood characteristics with poor oral health in older age: results from a crosssectional study of older british men |
| wrong population | Kinugawa (2022) | Association of poor dental status with eating alone: A cross-sectional Japan gerontological evaluation study among independent older adults. |
| wrong population | Neethu (2023) | Association of social network and social support with oral health among institutionalized elderly: A cross-sectional study in Bangalore city |
| wrong population | Mohamad Fuad (2020) | Association of sociodemographic factors and self-perception of health status on oral health-related quality of life among the older persons in Malaysia. |
| wrong outcome or exposure | Jung (2011) | Association of total tooth loss with socio-behavioural health indicators in Korean elderly. |
| wrong population | Gonçalves (2016) | Associations Between Caries Experience, Nutritional Status, Oral Hygiene, and Diet in a Multigenerational Cohort. |
| wrong population | Carlsson (2015) | Associations between dental anxiety, sense of coherence, oral health-related quality of life and health behavior-a national Swedish cross-sectional survey |
| cannot retrieve full text | Pengpid (2022) | Associations between edentulism and health indicators among older adults in India |
| wrong outcome or exposure | Srisilapanan (2016) | Associations between social inequality and tooth loss in a household sample of elderly Thai people aged ≥60 years old. |
| wrong population | Rosing (2019) | Associations between tooth loss, prostheses and self-reported oral health, general health, socioeconomic position and satisfaction with life |
| cannot retrieve full text | Pitter (1986) | A study of factors affecting the dental care of an elderly population in Bath Health District. |
| wrong population | Hwang (2023) | A Study on the Relationship between Food Security and the Number of Remaining Teeth in Korean Adults: The Korea National Health and Nutrition Examination Survey (KNHANES VII), 2016–2018 |
| wrong outcome or exposure | Edman (2018) | Attitudes to dental care, Sweden 2003-2013, and clinical correlates of oral health-related quality of life in 2013 |
| wrong outcome or exposure | Shanahan (2017) | Barriers to dental attendance in older patients. |
| wrong outcome or exposure | Fiske (1990) | Barriers to dental care in an elderly population resident in an inner city area. |
| wrong population | Osmari (2016) | Behaviour of the Elderly with Regard to Hygiene Procedures for and Maintenance of Removable Dentures. |
| wrong population | Emami (2010) | Better oral health related quality of life: type of prosthesis or psychological robustness? |
| wrong population | Kang (2020) | Bidirectional relations between cognitive function and oral health in ageing persons: A longitudinal cohort study |
| wrong population | Oliveira (2023) | Binge drinking and oral health-related quality of life in older adults: Socioeconomic position matters. |
| wrong population | Pereira (2019) | Budgetary Impact of Oral Rehabilitation with Complete Dentures: An Economical Evaluation from São Paulo State, Brazil |
| wrong publication type | Chicote (2019) | Care aide abilities in oral care delivery and seniors' oral health outcomes. |
| wrong outcome or exposure | Chalmers (2002) | Caries incidence and increments in community-living older adults with and without dementia |
| wrong outcome or exposure | Powell (1999) | Caries prevention in a community-dwelling older population |
| wrong population | Gülcan (2014) | Change in Oral Impacts on Daily Performances (OIDP) with increasing age: testing the evaluative properties of the OIDP frequency inventory using prospective data from Norway and Sweden. |
| wrong outcome or exposure | Griffin (2019) | Changes in Older Adults' Oral Health and Disparities: 1999 to 2004 and 2011 to 2016. |
| wrong population | Martins (2008) | Characteristics associated with use of dental services by dentate and edentulous elders: the SB Brazil Project. |
| wrong population | Chavers (2004) | Chronic oral disadvantage, a measure of long-term decrements in oral health-related quality of life |
| cannot retrieve full text | Zhao (2011) | Clinical and socio-demographic factors influencing the oral health-related quality of life of Chinese elders. |
| wrong population | Tellez (2015) | Clinical Management of Anxiety and Access to Health Care |
| wrong population | Saito (2013) | Cognitive function and number of teeth in a community-dwelling population in Japan |
| wrong outcome or exposure | Miranda (2012) | Cognitive impairment, the Mini-Mental State Examination and socio-demographic and dental variables in the elderly in Brazil. |
| wrong outcome or exposure | Kailembo (2016) | Common risk factors and edentulism in adults, aged 50 years and over, in China, Ghana, India and South Africa: results from the WHO Study on global AGEing and adult health (SAGE). |
| wrong outcome or exposure | Kim (2018) | Comparison of oral health status and daily nutrient intake between elders who live alone and elders who live with family: Based on the Korean National Health and Nutrition Examination Survey (KNHANES VI) (2013-2015). |
| cannot retrieve full text | Al-Dwairi (2010) | Complete edentulism and socioeconomic factors in a Jordanian population |
| wrong population | Dalazen (2016) | Contextual and Individual Factors Influencing Periodontal Treatment Needs by Elderly Brazilians: A Multilevel Analysis. |
| wrong publication type | Barbato (2015) | Contextual socioeconomic determinants of tooth loss in adults and elderly: a systematic review. |
| cannot retrieve full text | Miller (1994) | Correlates of tooth loss in a Canadian adult population |
| wrong outcome or exposure | Krasowski (2021) | Costs for statutorily insured dental services in older Germans 2012–2017 |
| wrong outcome or exposure | Ambjørnsen (1986) | Decayed, missing, and filled teeth among elderly people in a Norwegian municipality. |
| wrong population | Yuan (2024) | Decomposition Analysis of the Prevalence of Denture Use Between Rural and Urban Older Individuals With Edentulism in China: Cross-Sectional Study. |
| wrong population | Akinlotan (2024) | Delayed oral health care due to cost among US adults with diabetes, 2018-2019 |
| wrong outcome or exposure | Ringland (2004) | Demographic and socio-economic factors associated with dental health among older people in NSW. |
| cannot retrieve full text | Kandelman (1982) | Demographic, social and cultural factors influencing the elderly to seek dental treatment. |
| cannot retrieve full text | de Moura (2006) | Demographic, socio-economic, behavioural and clinical variables associated with caries activity |
| wrong population | Hägglin (1996) | Dental anxiety among middle-aged and elderly women in Sweden. A study of oral state, utilisation of dental services and concomitant factors |
| wrong population | Janardhanan (2011) | Dental care and associated factors among older adults with schizophrenia. |
| wrong population | Arcury (2012) | Dental care utilization among North Carolina rural older adults. |
| wrong population | Henriksen (2004) | Dental caries among the elderly in Norway |
| wrong publication type | Jain (2007) | Dental caries and social deprivation |
| wrong population | Arpin (2008) | Dental caries, problems perceived and use of services among institutionalized elderly in 3 regions of Quebec, Canada. |
| wrong population | Palmqvist (2000) | Dental conditions in middle-aged and older people in Denmark and Sweden: a comparative study of the influence of socioeconomic and attitudinal factors. |
| wrong population | Strayer (1993) | Dental health among homebound elderly. |
| wrong population | Haliti (2022) | Dental Health and Quality of Life in 117 Patients from Kosovo, Aged 6-80 Years, Evaluated Using the Dental Impact on Daily Living (DIDL) Questionnaire and the Oral Health-Related Quality of Life (OHRQoL) Questionnaire. |
| wrong population | Selikowitz (1986) | Dental health behavior in a migrant perspective: use of dental services of Pakistani immigrants in Norway |
| wrong population | Pizarro (2006) | Dental health differences by social class in home-dwelling seniors of Barcelona, Spain. |
| wrong publication type | Drugan (2011) | Dental health in the United Kingdom and influencing variables |
| wrong outcome or exposure | Stubbs (2002) | Dental screening of older adults living in residential aged care facilities in Perth. |
| wrong population | Simon (2021) | Dental services use before and after inpatient admission among privately insured adults in the United States |
| wrong population | Adams (2004) | Dental visits in older Western Australians: A comparison of urban, rural and remote residents |
| wrong population | dos Santos (2010) | Denture stomatitis and its risk indicators in south Brazilian older adults. |
| wrong population | de Lima Saintrain (2021) | Dependence in instrumental activities of daily living and its implications for older adults’ oral health |
| wrong population | Hugo (2012) | Depressive symptoms and untreated dental caries in older independently living South Brazilians. |
| wrong outcome or exposure | Albandar (1999) | Destructive periodontal disease in adults 30 years of age and older in the United States, 1988-1994 |
| wrong population | Schuller (2023) | Determinants and barriers for visiting a dental clinic among (frail) older individuals. |
| wrong population | Spinler (2021) | Determinants of dental treatment avoidance: findings from a nationally representative study. |
| wrong population | Kuthy (1996) | Determinants of dental user groups among an elderly, low-income population |
| wrong population | Aldosari (2022) | Diabetes mellitus and its association with dental caries, missing teeth and dental services utilization in the US adult population: Results from the 2015–2018 National Health and Nutrition Examination Survey |
| wrong population | Borges-Yañez (2004) | Dietary fiber intake and dental health status in urban, urban-marginal, and rural communities in central Mexico |
| wrong population | Ettinger (1973) | Diet, nutrition, and masticatory ability in a group of elderly edentulous patients. |
| wrong population | Hunt (1995) | Differences between racial groups in the impact of oral disorders among older adults in North Carolina. |
| wrong outcome or exposure | Preciado (2012) | Differences in impact of patient and prosthetic characteristics on oral health-related quality of life among implant-retained overdenture wearers. |
| wrong population | MacEntee (2012) | Discussions on oral health care among elderly Chinese immigrants in Melbourne and Vancouver. |
| wrong population | Albandar (2024) | Disparities and social determinants of periodontal diseases |
| wrong population | Espinoza (2013) | Disparities in aspects of oral-health-related quality of life among Chilean adults |
| wrong population | Quandt (2009) | Disparities in oral health status between older adults in a multiethnic rural community: the rural nutrition and oral health study. |
| wrong population | Wu (2025) | Disparities of Delayed Dental Visits and Severe Tooth Loss Among Older Native Hawaiians and Other Pacific Islanders in Hawai'i: A Cross-Sectional Study of Behavioral Risk Factor Surveillance System Data from 2012 to 2020. |
| wrong population | Moeller (2019) | Diversity in the use of specialized dental services by older adults in the United States. |
| wrong outcome or exposure | Thorstensson (2009) | Does oral health say anything about survival in later life? Findings in a Swedish cohort of 80+ years at baseline. |
| wrong population | Choi (2011) | Does regional socioeconomic context affect the dental caries experience? A multilevel study of Korean adults |
| wrong population | Allen (2024) | Does subsidizing the cost of care impact on dental attendance patterns among older adults? |
| cannot retrieve full text | Garms-Homolová (1982) | Do the elderly receive adequate dental care? Determinants of the use of dental services by urban dwellers aged between 60 and 90 - Part I. |
| wrong outcome or exposure | Schein (1999) | Dry eye and dry mouth in the elderly: A population-based assessment |
| wrong population | Kossioni (2011) | Eating habits in older people in Greece: the role of age, dental status and chewing difficulties. |
| wrong outcome or exposure | Ren (2017) | Edentulism and associated factors among community-dwelling middle-aged and elderly adults in China. |
| wrong population | Adams (2003) | Edentulism and associated factors in people 60 years and over from urban, rural and remote Western Australia. |
| wrong population | Jaafar (2020) | Edentulism and factors associated with community dwelling elderly in pasir puteh, Kelantan, Malaysia |
| wrong outcome or exposure | Gutiérrez (2024) | Edentulism and Individual Factors of Active Aging Framework in Colombia. |
| wrong population | Kiuchi (2023) | Education-related inequalities in oral health among older adults: Comparing Singapore and Japan. |
| wrong outcome or exposure | Li (2023) | Effects of Alzheimer's disease and related dementias on dental care usage and economic burden in older adults: a cross-sectional study |
| wrong outcome or exposure | Jung (2022) | Effects of Integrative Cognitive Function Improvement Program on Cognitive Function, Oral Health, and Mental Health in Older People: A Randomized Clinical Trial |
| wrong population | Madero-Cabib (2022) | Employment Trajectories Across the Life Course and Oral Health Among Older Persons in a Developing Country. |
| wrong population | Brigg (2022) | Enabling people with severe mental illness to overcome barriers to access dental treatment: a qualitative study applying COM-B framework analysis |
| wrong publication type | Burt (1992) | Epidemiology of dental diseases in the elderly |
| wrong population | Xu (2023) | Establishment of models to predict factors influencing periodontitis in patients with type 2 diabetes mellitus |
| wrong outcome or exposure | Shelley (2011) | Ethnic disparities in self-reported oral health status and access to care among older adults in NYC. |
| wrong population | Arcury (2013) | Ethnic variation in oral health and social integration among older rural adults |
| wrong outcome or exposure | Dabrowski (2025) | Evaluating changes in dental status among Polish older adults over a decade: A comparative analysis of PolSenior (2009) and PolSenior2 (2019) surveys. |
| wrong population | Schensul (2022) | Evaluating Cognitive/Emotional and Behavioral Mediators of Oral Health Outcomes in Vulnerable Older Adults. |
| wrong population | Bof de Andrade (2025) | Evaluating socioeconomic inequalities in self-rated oral health and its contributing factors in Brazilian older adults. |
| wrong population | Wu (2022) | Evaluation and influencing factors of oral health-related quality of life of middle-aged and elderly in Chengdu. |
| wrong publication type | Cañaveral (2015) | Evaluation of the cognitive-functional factors and the state of oral health in adults aged between 65-75 years and 75 years and above. An epidemiologic multicentric transversal study |
| wrong population | Curtis (2021) | Everyday experiences of people living with dementia and their carers relating to oral health and dental care. |
| wrong outcome or exposure | Koyama (2021) | Examining the associations between oral health and social isolation: A cross-national comparative study between Japan and England. |
| wrong population | Ghanbarzadegan (2024) | Examining the Effect of Income-Based Inequalities and Dental Service Provision on Dental Service Utilization among Older Australians: A Multiple Mediation Analysis. |
| wrong population | Wintch (2014) | Executive directors' perceptions of oral health care of aging adults in long-term care settings. |
| wrong outcome or exposure | Badewy (2025) | Exploring the association between dental insurance coverage and dental care utilization and oral health among elderly Ontarians. |
| wrong outcome or exposure | Amoak (2024) | Exploring the association of self-rated oral health with self-rated general and mental health among older adults in a resource-poor context: Insights for advancing Sustainable Development Goal 3. |
| wrong publication type | Pronk (2024) | Exploring the complex relationships between health behaviors, health outcomes, social vulnerability, regional cultures, and oral health |
| wrong publication type | Botkin (2025) | Exploring the National Health and Nutrition Examination Survey Data: Oral Health and Activities of Daily Living |
| wrong population | Lee (2012) | Exploring the quality of life after denture-wearing within elders in Kaohsiung. |
| wrong population | Lu (2021) | Exploring the reciprocal relationship between cognitive function and edentulism among middle-aged and older adults in China |
| wrong population | Suksan (2022) | Factors Affecting Oral Health Care Behavior of the Elderly Ethnic Groups in Rural Areas |
| wrong population | Kranjčić (2014) | Factors affecting oral health-related quality of life among elderly croatian patients |
| wrong outcome or exposure | Weintraub (2019) | Factors Associated With Becoming Edentulous in the US Health and Retirement Study. |
| wrong outcome or exposure | Gellacic (2016) | Factors associated with deterioration of self-rated chewing ability among adults aged 60 years and older over a 6-year period. |
| wrong outcome or exposure | Melo (2016) | Factors associated with negative self-perception of oral health among institutionalized elderly. |
| wrong outcome or exposure | Ekanayke (2005) | Factors associated with perceived oral health status in older individuals. |
| wrong outcome or exposure | Chae (2017) | Factors associated with perceived oral health status in older individuals. |
| wrong publication type | Tu (2023) | Factors associated with regular dental attendance by aged adults: A systematic review. |
| wrong population | Fagundes (2022) | Factors associated with self-perceived oral health in different age groups. |
| wrong population | Chantaraboot (2022) | Factors Associated with Self-reported Oral Health Among Community-Dwelling Older Adults in a Rural Province of Thailand |
| wrong outcome or exposure | Park (2025) | Factors associated with subjective oral health among older adults aged 65 and over living alone: the role of social capital. |
| wrong population | Gomes Filho (2020) | Factors associated with the need for a complete denture in one arch or both arches among the elderly population. |
| wrong population | A-Dan (2011) | Factors associated with the oral health-related quality of life in elderly persons in dental clinic: validation of a Mandarin Chinese version of GOHAI. |
| wrong population | Ferreira (2013) | Factors associated with the use of dental services by elderly Brazilians. |
| wrong outcome or exposure | Gondro (2024) | Factors associated with the use of oral health care services among seniors in Canada. |
| wrong population | Gabiec (2022) | Factors Associated with Tooth Loss in General Population of Bialystok, Poland. |
| cannot retrieve full text | Lourenço (2024) | Factors Associated with Total Edentulism in Older Adults and Their Impact on the Self-Perception of Oral Health and Food. |
| wrong population | Soares (2021) | Factors associated with utilisation of dental services by the elders from São Paulo, Brazil. |
| wrong population | Natto (2014) | Factors contributing to tooth loss among the elderly: A cross sectional study. |
| wrong outcome or exposure | MacEntee (1987) | Factors influencing oral health in long term care facilities. |
| wrong population | Choi (2021) | Factors influencing oral health-related quality of life in older adults in rural areas: Oral dryness and oral health knowledge and behavior |
| wrong outcome or exposure | Moon (2020) | Factors influencing self-rated oral health in elderly people residing in the community: Results from the korea community health survey, 2016 |
| wrong outcome or exposure | Leake (1996) | Factors influencing the amount and type of dental services received by older adults in four municipalities in Ontario, Canada. |
| wrong population | Borrell (2003) | Factors influencing the effect of race on established periodontitis prevalence |
| wrong population | Woods (2009) | Factors influencing the need for dental care amongst the elderly in the Republic of Ireland. |
| wrong population | Hariyani (2020) | Factors influencing the utilization of dental services in East Java, Indonesia |
| wrong outcome or exposure | Kim (2016) | Factors related to number of present teeth in Korean elderly adults aged 55-84 years. |
| wrong outcome or exposure | Andrade (2012) | Factors related to poor self-perceived oral health among community-dwelling elderly individuals in São Paulo, Brazil. |
| wrong outcome or exposure | Ghazal (2015) | Factors related to receipt of dental care in Eastern Iowa nursing facilities. |
| wrong outcome or exposure | Vehkalahti (1996) | Factors related to the non-attendance in a clinical oral health study on the home-dwelling old elderly |
| wrong population | Ando (2013) | Factors related to tooth loss among community-dwelling middle-aged and elderly Japanese men. |
| wrong publication type | Glassman (2005) | Financing oral health services for people with special needs: projecting national expenditures. |
| wrong population | Lee (2025) | Food insecurity and its relationship to mental health and oral health in older adults. |
| wrong population | Janssens (2023) | Frailty as a determinant of dental attendance among community-dwelling older adults. |
| wrong population | Brennan (2010) | Fruit and vegetable consumption among older adults by tooth loss and socio-economic status. |
| wrong population | Bomfim (2018) | Functional dentition and associated factors in Brazilian elderly people: A multilevel generalised structural equation modelling approach. |
| wrong outcome or exposure | Dolan (1998) | Functional health and dental service use among older adults |
| wrong outcome or exposure | Chi (2013) | Gender-stratified models to examine the relationship between financial hardship and self-reported oral health for older US men and women. |
| wrong population | Skośkiewicz-Malinowska (2021) | Gender-wise comparison of oral health quality of life and its relationship with oral health parameters among elderly from Wroclaw, south-west Poland |
| wrong outcome or exposure | Brennan (2018) | General health and well-being among primary care patients aged 75+ years: Associations with living conditions, oral health and dependency. |
| wrong population | Ozkan (2011) | General health, dental status and perceived dental treatment needs of an elderly population in Istanbul. |
| wrong population | Locker (2002) | General health status and changes in chewing ability in older Canadians over seven years. |
| wrong population | Sukumar (2024) | Geriatric Oral Health and Quality of Life Among the Indigenous Irula Tribes of Tamil Nadu. |
| wrong publication type | Luhanga (2001) | Geriatric oral health issues in Africa: Tanzanian perspective. |
| wrong outcome or exposure | Matsuda (2021) | Has the COVID-19 pandemic influenced the oral health and nutritional status of elderly patients with digestive cancer?: A retrospective study in Fukui, Japan. |
| cannot retrieve full text | Beck (1988) | Identification of high caries risk adults: attitudes, social factors and diseases. |
| wrong population | Jiang (2025) | Immigrant Status, Social Support From Children, and Resilience for Older Parents' Perceived Oral Health Among Chinese Americans. |
| wrong population | Amaral (2021) | Impact of adopting different socioeconomic indicators in older adults' oral health research. |
| wrong population | Hsu (2013) | Impact of oral health behaviours and oral habits on the number of remaining teeth in older Taiwanese dentate adults |
| wrong publication type | Yu (2024) | Impact of oral health literacy on oral health behaviors and outcomes among the older adults: a scoping review. |
| wrong population | Biazevic (2004) | Impact of oral health on quality of life among the elderly population of Joaçaba, Santa Catarina, Brazil. |
| wrong population | Dehmoobadsharifabadi (2018) | Impact of public dental care spending and insurance coverage on utilization disparities among Canadian jurisdictions. |
| wrong population | Machado (2020) | Impact of Seniors Centers on oral health-related quality of life of older adults. |
| wrong publication type | Serpa (2015) | Impact of social determinants in oral health in older patients admitted in a medical ward of a general hospital |
| wrong publication type | Dahlan (2019) | Impact of social support on oral health among immigrants and ethnic minorities: A systematic review |
| wrong population | Bhochhibhoya (2022) | Impact of sociodemographic factors, duration of edentulism, and medical comorbidities on the mental attitudes of individuals with complete edentulism. |
| wrong population | Shah (2003) | Impact of socio-demographic variables, oral hygiene practices and oral habits on periodontal health status of Indian elderly: a community-based study. |
| wrong population | Shah (2004) | Impact of socio-demographic variables, oral hygiene practices, oral habits and diet on dental caries experience of Indian elderly: a community-based study. |
| wrong publication type | Naito (2015) | Impact of socio-economic factors on oral health related quality of life in Japanese population |
| wrong population | Vysniauskaite (2009) | Impacts of toothbrushing frequency on periodontal findings in a group of elderly Lithuanians. |
| wrong population | Cooray (2021) | Importance of socioeconomic factors in predicting tooth loss among older adults in Japan: Evidence from a machine learning analysis. |
| wrong population | Haugejorden (2003) | Incidence and predictors of self-reported tooth loss in a representative sample of Norwegian adults |
| wrong outcome or exposure | Locker (1996) | Incidence of and risk factors for tooth loss in a population of older Canadians. |
| wrong outcome or exposure | Brown (1994) | Incidence of attachment loss in community-dwelling older adults. |
| wrong publication type | Scannapieco (2024) | Inclusion of Dental Services in Medicare to Improve Oral and General Health for Older Americans. |
| wrong population | Kailembo (2018) | Income and wealth as correlates of socioeconomic disparity in dentist visits among adults aged 20 years and over in the United States, 2011-2014 |
| wrong outcome or exposure | Aida (2011) | Income inequality, social capital and self-rated health and dental status in older Japanese. |
| wrong population | Lislt (2012) | Income-related inequalities in chewing ability of Europeans aged 50 and above. |
| wrong population | Raittio (2015) | Income-related inequality and inequity in the use of dental services in Finland after a major subsidization reform |
| wrong outcome or exposure | Islas-Granillo (2019) | Indicators of oral health in older adults with and without the presence of multimorbidity: A cross-sectional study |
| wrong population | Baldani (2010) | Individual determinants of dental care utilization among low-income adult and elderly individuals. |
| wrong population | Badr (2020) | Inequalities in Untreated Root Caries and Affordability of Dental Services among Older American Adults. |
| wrong population | Somkotra (2013) | Inequality in oral health-care utilisation exists among older Thais despite a universal coverage policy. |
| wrong population | Raittio (2015) | Inequality in oral health-related quality of life before and after a major subsidization reform |
| wrong population | Wamala (2006) | Inequity in access to dental care services explains current socioeconomic disparities in oral health: The Swedish National Surveys of Public Health 2004-2005 |
| wrong population | Hiramatsu (2006) | Influence of acculturation on self-perceived oral health among Japanese-Brazilian elderly |
| cannot retrieve full text | Palmqvist (2001) | Influence of dental care systems on dental status. A comparison between two countries with different systems but similar living standards |
| wrong outcome or exposure | Brochier (2018) | Influence of dental factors on oropharyngeal dysphagia among recipients of long-term care. |
| wrong outcome or exposure | Woo (2008) | Influence of different health-care systems on health of older adults: a comparison of Hong Kong, Beijing urban and rural cohorts aged 70 years and older. |
| wrong population | Binmogren (2024) | Influence of disabilities on state-level disparity in dental care utilization and tooth loss: Findings from the 2020 Behavioral Risk Factor Surveillance System |
| wrong population | de Lucena (2021) | Influence of socioeconomic status on oral disease burden: a population-based study. |
| wrong population | Stavreva (2015) | Influence of the Ethnic Affiliation, Level of Education and Place of Living on Oral Health at Geriatric Population with Total and Partial Dentures in Republic of Macedonia. |
| wrong population | McGrath (2002) | Influences of social support on the oral health of older people in Britain. |
| wrong population | Ruas (2020) | Integrality of care for hemodialysis patient in Brazil: an analysis of access to dental care |
| wrong population | York (2024) | Integrating social determinants into routine dental care for a person-centered approach |
| wrong publication type | Gomez-Rossi (2020) | Interventions to improve oral health of older people: A scoping review. |
| wrong population | Shen (2018) | Investigating social inequalities in older adults' dentition and the role of dental service use in 14 European countries. |
| wrong outcome or exposure | Hjertstedt (2014) | Investigating the impact of a community-based geriatric dentistry rotation on oral health literacy and oral hygiene of older adults. |
| wrong population | Wang (2014) | Investigating tooth loss and associated factors among older Taiwanese adults. |
| wrong population | Hamano (2017) | Is Accessibility to Dental Care Facilities in Rural Areas Associated with Number of Teeth in Elderly Residents? |
| wrong population | Crocombe (2012) | Is poor access to dental care why people outside capital cities have poor oral health? |
| wrong outcome or exposure | Crocombe (2012) | Is self interdental cleaning associated with dental plaque levels, dental calculus, gingivitis and periodontal disease? |
| wrong outcome or exposure | Rouxel (2015) | Is Social Capital a Determinant of Oral Health among Older Adults? Findings from the English Longitudinal Study of Ageing. |
| wrong population | Aida (2016) | Is social network diversity associated with tooth loss among older Japanese adults? |
| wrong outcome or exposure | Hakeem (2019) | Is there socioeconomic inequality in periodontal disease among adults with optimal behaviours |
| wrong publication type | Park (2014) | Late-life depression and oral hygiene in U.S. Older adults |
| cannot retrieve full text | Locker (1997) | Life circumstances, lifestyles and oral health among older Canadians |
| wrong population | Almoznino (2015) | Lifestyle and dental attendance as predictors of oral health-related quality of life |
| wrong population | Salih (2023) | Linking the demographic, socio-economic and oral health status to oral health-related quality of life of the sudanese older adults: a cross sectional study. |
| wrong population | Singh (2020) | Loneliness and disability as predictors of oral diseases among 2 groups of older adults in central India. |
| wrong outcome or exposure | Iwasaki (2015) | Longitudinal relationship between metabolic syndrome and periodontal disease among Japanese adults aged ≥70 years: the Niigata Study. |
| wrong outcome or exposure | Weyant (2004) | Medical and cognitive correlates of denture wearing in older community-dwelling adults. |
| wrong population | Marsk (2024) | Metabolic surgery and oral health: A register-based study |
| wrong population | Suwanprasit (2021) | Metabolic syndrome and severe periodontitis were associated in Thai adults: A cross-sectional study |
| wrong population | Holde (2016) | Methods and background characteristics of the TOHNN study: a population-based study of oral health conditions in northern Norway |
| wrong outcome or exposure | Liu (2025) | Migrant-Local Differences in the Relationship Between Oral Health, Social Support, and Loneliness Among Older Adults in Weifang, China: Cross-Sectional Study. |
| wrong outcome or exposure | Ferry (2024) | Mind the gap: an administrative data analysis of dental treatment outcomes and severe mental illness |
| wrong population | Lee (2016) | National dental policies and socio-demographic factors affecting changes in the incidence of periodontal treatments in Korean: A nationwide population-based retrospective cohort study from 2002-2013 |
| wrong population | Głowacka (2019) | Needs for gerodontological treatment in the elderly living in lower silesia |
| wrong population | Mao (2020) | Neighborhood Cohesion and Oral Health Problems Among Older Chinese American Immigrants: Does Acculturation Make a Difference? |
| wrong outcome or exposure | Lang (2008) | Neighbourhood deprivation and dental service use: a cross-sectional analysis of older people in England. |
| wrong publication type | Shinsho (2001) | New strategy for better geriatric oral health in Japan: 80/20 movement and Healthy Japan 21. |
| wrong population | Susin (2005) | Occurrence and risk indicators of increased probing depth in an adult Brazilian population |
| wrong outcome or exposure | Kaur (2024) | Occurrence of Depression and its Association with Oral Health in Elderly People Living in Old Age Homes of Mathura |
| wrong population | Saintrain (2018) | Older adults' dependence in activities of daily living: Implications for oral health. |
| wrong population | Maxwell (2014) | Oral health among residents of publicly supported housing in Boston |
| wrong outcome or exposure | Singh (2015) | Oral health among the elderly in 7 Latin American and Caribbean cities, 1999-2000: a cross-sectional study. |
| wrong population | Aarabi (2018) | Oral health and access to dental care - a comparison of elderly migrants and non-migrants in Germany. |
| wrong population | Slack-Smith (2010) | Oral health and access to dental care: a qualitative investigation among older people in the community. |
| wrong publication type | Wong (2019) | Oral health and its associated factors among older institutionalized residents—a systematic review |
| cannot retrieve full text | Palmqvist (1986) | Oral health and socio-economic factors in a Swedish county population aged 65 and over |
| wrong population | Gianopoulos (2014) | Oral health assessment in the San Blas and Santa Ana populations of Nicaragua |
| wrong outcome or exposure | Hugo (2007) | Oral health behaviour and socio-demographic profile of subjects with Alzheimer's disease as reported by their family caregivers |
| wrong publication type | Chen (2015) | Oral health care for older adults with serious illness: When and how? |
| wrong publication type | Nghayo (2024) | Oral health community engagement programs for rural communities: A scoping review. |
| wrong outcome or exposure | Schroeder (2020) | Oral health condition and the use of dental services among the older adults living in the rural area in the south of Brazil. |
| cannot retrieve full text | Duván (2025) | Oral health conditions and nutritional, functional and cognitive status - An approach to the institutionalized older adult |
| wrong population | Ji (2025) | Oral Health Differences Between Middle-Aged and Older Adults in South Korea. |
| wrong publication type | Le (2017) | Oral Health Disparities and Inequities in Asian Americans and Pacific Islanders |
| wrong publication type | Palma (2025) | Oral Health Inequalities and Functional Frailty in Older Adults: A Systematic Review and Meta-Analysis of Associated Factors |
| wrong population | Chari (2022) | Oral health inequality in Canada, the United States and United Kingdom |
| wrong outcome or exposure | Jamieson (2024) | Oral health inequities over time among older adults of different racial/ethnic backgrounds: a comparative decomposition analysis across Australia and the United States. |
| wrong publication type | Taani (2004) | Oral health in Jordan. |
| wrong population | Lee (2016) | Oral Health in Low-Income Older Adults in Korea. |
| wrong publication type | Protudjer (2020) | Oral health in rheumatoid arthritis: Listening to patients |
| wrong publication type | Simons (1999) | Oral health of elderly occupants in residential homes. |
| wrong outcome or exposure | Carneiro (2005) | Oral health of institutionalized elderly in the eastern zone of São Paulo, Brazil, 1999 |
| wrong population | Kim (2024) | Oral health of older Koreans by region and family status in 2020-2021. |
| wrong population | Teng (2011) | Oral health of psychiatric inpatients: A survey of central Taiwan hospitals |
| wrong population | Ribeiro (2012) | Oral health of the elderly with Alzheimer's disease. |
| cannot retrieve full text | Palmqvist (1986) | Oral health patterns in a Swedish county population aged 65 and above. |
| wrong outcome or exposure | Andrade (2019) | Oral health profile among community-dwellingelderly and its association with self-rated oral health. |
| cannot retrieve full text | Zhou (2012) | Oral health related quality of life among older adults in Central China. |
| wrong outcome or exposure | Dahl (2011) | Oral health-related quality of life and associated factors in Norwegian adults. |
| wrong outcome or exposure | Silva (2015) | Oral health-related quality of life and associated factors in Southern Brazilian elderly. |
| wrong population | Kishi (2015) | Oral health-related quality of life and related factors among residents in a disaster area of the Great East Japan Earthquake and giant tsunami |
| wrong population | Perea (2013) | Oral health-related quality of life in complete denture wearers depending on their socio-demographic background, prosthetic-related factors and clinical condition. |
| wrong population | Reisine (2021) | Oral health-related quality of life outcomes in a randomized clinical trial to assess a community-based oral hygiene intervention among adults living in low-income senior housing |
| wrong population | Wei (2024) | Oral health self-management barriers among rural older adults in Guangxi, China: A qualitative study. |
| wrong publication type | Lauritano (2021) | Oral health status among migrants from middle-and low-income countries to europe: A systematic review |
| cannot retrieve full text | Mariño (2011) | Oral health status and inequalities among ambulant older adults living in central Chile. |
| wrong outcome or exposure | Carter (2004) | Oral health status and oral treatment needs of dependent elderly people in Christchurch |
| wrong population | Gaio (2012) | Oral health status in elders from South Brazil: a population-based study. |
| wrong outcome or exposure | Balaganesh (2022) | Oral Health Status, Needs, Utilization and Barriers in Utilization of Dental Health Services among Rural Population |
| wrong population | Leung (2008) | Oral health status of low-income, middle-aged to elderly Hong Kong Chinese with type 2 diabetes mellitus. |
| wrong population | Panapasa (2003) | Oral health status of older adults in Fiji: 1985-1999. |
| wrong outcome or exposure | Bush (2010) | Oral health status of older adults in Kentucky: results from the Kentucky Elder Oral Health Survey. |
| cannot retrieve full text | Karkazis (1993) | Oral health status, treatment needs and demands of an elderly institutionalised population in Athens. |
| wrong population | Petrovsky (2019) | Oral Health Symptoms and Cognitive Function Among US Community-Dwelling Chinese Older Adults. |
| wrong outcome or exposure | Cohen (2006) | Oral hygiene for the institutionalized elderly |
| wrong population | Åstrøm (2006) | Oral Impacts on Daily Performance in Norwegian adults: The influence of age, number of missing teeth, and socio-demographic factors |
| wrong population | Ostberg (2009) | Oral impacts on daily performances: associations with self-reported general health and medication. |
| wrong outcome or exposure | Fløystrand (1982) | Oral status and acceptance of dental services among some elderly persons in Oslo. |
| wrong population | Kiuchi (2022) | Oral Status and Dementia Onset: Mediation of Nutritional and Social Factors. |
| wrong population | Abbas (2023) | Oral status and homebound status: A 6-year bidirectional exploratory prospective cohort study |
| wrong publication type | Ruiz-Roca (2021) | Oral status of older people in medium to long-stay health and social care setting: a systematic review. |
| wrong publication type | Yadav (2020) | Palliative dental care: Ignored dimension of dentistry amidst COVID-19 pandemic |
| wrong population | Menegazzo (2023) | Pathways that explain racial differences on edentulism among older adults: 2019 Brazil National Health Survey. |
| wrong population | Mao (2019) | Perceived Stress, Social Support, and Dry Mouth Among US Older Chinese Adults. |
| wrong population | An (2024) | Perceptions and experience of rural older people in oral health management in China: a qualitative study. |
| wrong population | Lorenzo (2015) | Periodontal conditions and associated factors among adults and the elderly: findings from the first National Oral Health Survey in Uruguay. |
| wrong publication type | Edman (2022) | Periodontal disease and factors associated with disease progression: A 10-year longitudinal study in an elderly population |
| wrong outcome or exposure | de Miguel-Infante (2019) | Periodontal disease in adults with diabetes, prevalence and risk factors. Results of an observational study |
| wrong population | Oliver (1998) | Periodontal diseases in the United States population |
| wrong population | Morales-Ruiz (2023) | Periodontal health of a low socioeconomic level population in Yucatan (Mexico): A cross-sectional study |
| wrong outcome or exposure | MacEntee (1991) | Predicting concerns for the mouth among institutionalized elders. |
| wrong outcome or exposure | Andersen (2025) | Prediction of oral diseases in care dependent older people. |
| wrong outcome or exposure | Starr (2008) | Predictors and correlates of edentulism in the healthy old people in Edinburgh (HOPE) study. |
| wrong population | Gonda (2013) | Predictors of multiple tooth loss among socioculturally diverse elderly subjects. |
| wrong outcome or exposure | Diehnelt (1990) | Predictors of oral health behaviors among elderly Japanese Americans. |
| wrong outcome or exposure | Limpuangthip (2019) | Predisposing and enabling factors associated with public denture service utilization among older Thai people: a cross-sectional population-based study. |
| wrong outcome or exposure | Lin (2025) | Prevalence and associated factors of denture use among older adults living in rural and urban areas of China: a national cross-sectional study. |
| cannot retrieve full text | Peltzer (2022) | Prevalence and correlates of oral health problems among a nationally representative sample of older adults in India, 2017-2018 |
| wrong outcome or exposure | Marcus (1994) | Prevalence and demographic correlates of tooth loss among the elderly in the United States. |
| cannot retrieve full text | Li (2007) | Prevalence and related factors of periodontitis in community-dwelling Chinese with diabetes |
| wrong population | Srivastava (2013) | Prevalence of dental caries and periodontal diseases, and their association with socio-demographic risk factors among older persons in Delhi, India: a community-based study. |
| wrong population | Ravindranath (2025) | Prevalence of Edentulism, Associated Factors, Perceived Treatment Needs and Barriers for Dental Care among the Elderly in a Rural Area of Puducherry, India: A Community-based Mixed-method Study |
| wrong outcome or exposure | Haikola (2008) | Prevalence of edentulousness and related factors among elderly Finns. |
| wrong outcome or exposure | Peltzer (2014) | Prevalence of loss of all teeth (edentulism) and associated factors in older adults in China, Ghana, India, Mexico, Russia and South Africa. |
| wrong population | Iwasaki (2024) | Prevalence of oral frailty and its association with dietary variety, social engagement, and physical frailty: Results from the Oral Frailty 5-Item Checklist. |
| wrong outcome or exposure | Mozafari (2012) | Prevalence of oral mucosal lesions in institutionalized elderly people in Mashhad, Northeast Iran. |
| wrong population | Dias-da-Costa (2010) | Prevalence of poor self-rated mastication and associated factors in Brazilian elderly. |
| wrong outcome or exposure | Randolph (2001) | Prevalence of tooth loss and dental service use in older Mexican Americans. |
| wrong population | Deguchi (2017) | Preventable Tooth Loss in Hawai'i: The Role of Socioeconomic Status, Diabetes, and Dental Visits |
| wrong population | Zini (2016) | Professional occupation and the number of teeth retained among older adults aged 50 and above. |
| wrong population | Teixeira (2016) | Prospective study on tooth loss in a cohort of dentate elderly. |
| wrong population | Maupomé (1998) | Prosthodontic profiles relating to economic status, social network, and social support in an elderly population living independently in Canada. |
| wrong population | Song (2023) | Psychosocial factors, dentist-patient relationships, and oral health-related quality of life: a structural equation modelling |
| cannot retrieve full text | Schou (1991) | Qualitative study of oral health norms and behaviour among elderly people in Scotland. |
| wrong outcome or exposure | Weatherspoon (2016) | Racial and Ethnic Differences in Self-Reported Periodontal Disease in the Multi-Ethnic Study of Atherosclerosis (MESA). |
| wrong population | Chavers (2002) | Racial and socioeconomic disparities in oral disadvantage, a measure of oral health-related quality of life: 24-month incidence |
| cannot retrieve full text | Soh (1992) | Racial differences in perception of oral health and oral health behaviours in Singapore |
| wrong population | Bomfim (2021) | Racial inequities in tooth loss among older Brazilian adults: A decomposition analysis. |
| wrong population | Chiba (2009) | Recognition and behaviour of caregiver managers related to oral care in the community. |
| wrong outcome or exposure | Yamamoto (2024) | Regional Inequalities in Oral Frailty and Social Capital. |
| wrong population | Taira (2021) | Regional Inequality in Dental Care Utilization in Japan: An Ecological Study Using the National Database of Health Insurance Claims |
| wrong population | Ranta (1987) | Rehabilitation with removable dentures among the dentate population in Finland |
| wrong population | Syrjälä (2007) | Relationship between cognitive impairment and oral health: results of the Health 2000 Health Examination Survey in Finland. |
| wrong outcome or exposure | Hiltunen (2021) | Relationship between Fried's frailty phenotype and oral frailty in long-term care residents |
| wrong population | Hernández-Palacios (2015) | Relationship between gender, income and education and self-perceived oral health among elderly Mexicans. An exploratory study. |
| wrong population | Norlén (1991) | Relationship between general health, social factors and oral health in women at the age of retirement. |
| wrong outcome or exposure | Minagawa (2015) | Relationship between metabolic syndrome and periodontitis in 80-year-old Japanese subjects. |
| wrong population | de Andrade (2012) | Relationship between oral health-related quality of life, oral health, socioeconomic, and general health factors in elderly Brazilians. |
| wrong outcome or exposure | Hiroshimaya (2025) | Relationship between oral hypofunction and medical expenditure in older adults in Japan |
| wrong population | Davoglio (2016) | Relationship between Sense of Coherence and oral health in adults and elderly Brazilians. |
| wrong outcome or exposure | Cho (2018) | Relationship between the activities of daily living, oral health-related quality of life and dementia. |
| wrong population | Moriya (2013) | Relationships between higher-level functional capacity and dental health behaviors in community-dwelling older adults. |
| wrong population | Pien (2023) | Relationships between stroke prevalence, health literacy, and oral health-related quality of life in middle-aged and older adults: a national survey study. |
| wrong population | Singh (2024) | Risk factors and associated pathway for rural and urban unmet dental prosthetic needs among older adults in Central India. |
| wrong population | Borges-Yañez (2006) | Risk factors and prevalence of periodontitis in community-dwelling elders in Mexico. |
| wrong population | Saito (2019) | Risk factors for tooth loss in adult Japanese dental patients: 8020 Promotion Foundation Study |
| wrong population | Locker (1993) | Risk indicators and risk markers for periodontal disease experience in older adults living independently in Ontario, Canada. |
| wrong population | Hayes (2016) | Risk indicators associated with root caries in independently living older adults. |
| wrong population | Ribeiro (2015) | Risk indicators for tooth loss in Kiriri Adult Indians: a cross-sectional study |
| wrong outcome or exposure | Dolan (2001) | Risk indicators of edentulism, partial tooth loss and prosthetic status among black and white middle-aged and older adults. |
| wrong population | Christensen (2015) | Root caries, root surface restorations and lifestyle factors in adult Danes |
| wrong population | Hariyani (2018) | Root surface caries among older Australians. |
| wrong publication type | Shmarina (2021) | Salutogenic factors for oral health among older people: an integrative review connecting the theoretical frameworks of Antonovsky and Lalonde. |
| wrong outcome or exposure | Morita (2007) | Salutogenic factors that may enhance lifelong oral health in an elderly Japanese population. |
| wrong outcome or exposure | Liu (2024) | Self-care ability and associated factors in community-dwelling older adults living with oral frailty using the COM-B model. |
| wrong population | Matos (2006) | Self-rated oral health among Brazilian adults and older adults in Southeast Brazil: results from the SB-Brasil Project, 2003. |
| wrong population | Mejia (2014) | Self-rated oral health and oral health-related factors: The role of social inequality |
| wrong outcome or exposure | Neves-Guimaraes (2025) | Self-reported data validity for assessment of systemic and oral health as risk for dependency in old age: a cohort profile of elderly individuals in mid Sweden. |
| wrong outcome or exposure | Thomson (1996) | Self-reported dental status and treatment need among elderly people. |
| wrong outcome or exposure | Bassim (2020) | Self-reported oral health at baseline of the Canadian Longitudinal Study on Aging. |
| wrong outcome or exposure | Buhlin (2003) | Self-reported oral health, dental care habits and cardiovascular disease in an adult Swedish population |
| wrong population | Estrada (2018) | "Seniors only want respect": designing an oral health program for older adults. |
| wrong population | Bernabé (2010) | Sense of coherence and oral health in dentate adults: Findings from the Finnish Health 2000 survey |
| wrong population | Huntington (1999) | SES and oral health status in an elderly population. |
| wrong outcome or exposure | Thompson (2018) | Sinking teeth into dental health of older adults in Jamaica |
| cannot retrieve full text | Peltzer (2022) | Social and health determinants of edentulism among older adults in India |
| wrong outcome or exposure | Rouxel (2015) | Social capital and oral health among adults 50 years and older: Results from the English longitudinal study of ageing |
| wrong population | Moghaddam (2023) | Social cognitive predictors of regular dental visits and mouth self-examination behaviors among the elderly population: An application of the health action process approach model |
| wrong population | Liu (2025) | Social context matters: Neighborhood environment as a moderator of the longitudinal relationship between edentulism and cognitive function among older adults in the United States |
| wrong population | Gilbert (2003) | Social Determinants of Tooth Loss |
| wrong population | Liu (2014) | Social disparities in dentition status among American adults |
| wrong publication type | Navarro (2012) | Social disparity and oral health |
| wrong publication type | Wright (2015) | Social implications and workforce issues in the oral health of an ageing population. |
| wrong population | da Silva (2015) | Social Inequalities and the Oral health in Brazilian Capitals |
| wrong population | El Osta (2018) | Social inequalities in oral health in a group of older people in a Middle Eastern country: a cross-sectional survey. |
| cannot retrieve full text | Rickardsson (1989) | Social network and regular dental care utilisation in elderly men. Results from the population study "Men born in 1914", Malmö, Sweden. |
| wrong population | Takeuchi (2013) | Social Participation and Dental Health Status among Older Japanese Adults: A Population-Based Cross-Sectional Study |
| wrong publication type | Yoshida-Kohno (2024) | Social Relationships and Tooth Loss in Adults Aged 60 Years and Older: A Systematic Review and Meta-Analysis |
| wrong population | Laniado (2023) | Social support and oral health among working-age and older adults in the United States. |
| wrong population | Wu (2014) | Social stratification and tooth loss among middle-aged and older Americans from 1988 to 2004. |
| wrong population | Maida (2013) | Socio-behavioral predictors of self-reported oral health-related quality of life |
| wrong publication type | Roberto (2019) | Sociodemographic determinants of edentulism in the elderly population: A systematic review and meta-analysis. |
| cannot retrieve full text | Brown (1988) | Sociodemographic differences in tooth loss patterns in U.S. employed adults and seniors, 1985-86. |
| wrong population | de Oliveira (2013) | Socio-demographic factors and oral health conditions in the elderly: a population-based study. |
| wrong publication type | Azogui-Lévy (2012) | Sociodemographic factors associated with the dental health of persons with diabetes in France |
| wrong outcome or exposure | Åstrøm (2025) | Sociodemographic Inequalities in Oral Health-Related Quality of Life in Older Adults: 15 Years Follow-Up of the 1932 and 1942 Birth Cohorts in Sweden. |
| wrong population | Huang (2015) | Socioeconomic and racial/ethnic oral health disparities among US older adults: oral health quality of life and dentition. |
| wrong population | Li (2021) | Socio-Economic Disparities in Dental Health and Dental Care Utilisation Among Older Chinese. |
| wrong population | Bomfim (2018) | Socio-economic factors associated with periodontal conditions among Brazilian elderly people - Multilevel analysis of the SBSP-15 study. |
| wrong population | Elani (2012) | Socio-economic inequalities and oral health in Canada and the United States |
| wrong population | Ghorbani (2015) | Socioeconomic inequalities in oral health among adults in Tehran, Iran |
| wrong population | Fagundes (2021) | Socioeconomic inequalities in the use of dental services in Brazil: an analysis of the 2019 National Health Survey. |
| wrong outcome or exposure | Kailembo (2018) | Socioeconomic inequality in self-reported unmet need for oral health services in adults aged 50 years and over in China, Ghana, and India. |
| wrong population | Haugejorden (2008) | Socio-economic inequality in the self-reported number of natural teeth among Norwegian adults - An analytical study |
| wrong population | Frisk (2006) | Socio-economic risk indicators for apical periodontitis |
| wrong outcome or exposure | Parker (1999) | Socioeconomic status and health among elderly people in Sweden |
| wrong outcome or exposure | Nakahori (2019) | Socioeconomic status and remaining teeth in Japan: results from the Toyama dementia survey. |
| wrong population | Lyu (2024) | Socioeconomic Status and Tooth Loss Impact on Oral Health-Related Quality of Life in Chinese Elderly. |
| wrong publication type | Blau (1982) | Socioeconomic variations in dental status and behavior of today's elderly. |
| wrong outcome or exposure | Kossioni (1999) | Socio-medical condition and oral functional status in an older institutionalised population. |
| wrong outcome or exposure | Moreira (2011) | Spatial risk and factors associated with edentulism among elderly persons in Southeast Brazil. |
| wrong population | Nyyssōnen (1987) | Subjective need of removable denture treatment in Finnish adults |
| wrong outcome or exposure | Limo (2024) | Suboptimal Oral Health, Multimorbidity, and Access to Dental Care |
| wrong publication type | Osuh (2024) | Systematic review of oral health in slums and non-slum urban settings of Low and Middle-Income Countries (LMICs): Disease prevalence, determinants, perception, and practices |
| wrong publication type | Cianetti (2021) | Systematic review of the literature on dental caries and periodontal disease in socio-economically disadvantaged individuals |
| cannot retrieve full text | Almagro Céspedes (2011) | Temporomandibular joint dysfunction, disability and oral health in a community-dwelling elderly population. |
| wrong population | Moeller (2016) | The Association Between Income Inequality and Oral Health in Canada: A Cross-Sectional Study |
| wrong population | Delbari (2025) | The association between oral health and mild cognitive impairment among Iranian older adults: A cross-sectional study from Ardakan Cohort Study on Aging (ACSA) |
| wrong population | Ye (2024) | The association between oral health and mild cognitive impairment in community-dwelling older adults. |
| wrong publication type | Yun (2023) | The association between social isolation and oral health of community-dwelling older adults-A systematic review. |
| cannot retrieve full text | Locker (1992) | The burden of oral disorders in a population of older adults. |
| cannot retrieve full text | Shnider (2012) | The changing complexity of geriatric health care and the implications for oral health care for the individual and community. |
| wrong outcome or exposure | Heegaard (2011) | The Copenhagen Oral Health Senior Cohort: design, population and dental health. |
| wrong publication type | Liang (2025) | The current status and influencing factors of oral frailty in elderly populations: A scoping review. |
| cannot retrieve full text | Lutskaia (1995) | The demand for dental care among persons of older age groups |
| wrong population | Aida (2009) | The different effects of vertical social capital and horizontal social capital on dental status: a multilevel analysis. |
| wrong outcome or exposure | Wang (2022) | The effects of oral health and social support on health-related quality of life of migrant older with children in Weifang, China. |
| wrong population | Huber (1975) | The extent of the dependence of dental care and dental status from social class |
| wrong population | Persson (2004) | The impact of ethnicity, gender, and marital status on periodontal and systemic health of older subjects in the Trials to Enhance Elders' Teeth and Oral Health (TEETH). |
| wrong outcome or exposure | McMillan (2003) | The impact of oral disease among the institutionalized and non-institutionalized elderly in Hong Kong. |
| wrong outcome or exposure | Porter (2015) | The impact of oral health on the quality of life of nursing home residents |
| wrong population | Malicka (2022) | The impact of socioeconomic status, general health and oral health on Health-Related Quality of Life, Oral Health-Related Quality of Life and mental health among Polish older adults. |
| wrong publication type | Stennett (2022) | The impact of the COVID-19 pandemic on oral health inequalities and access to oral healthcare in England. |
| wrong outcome or exposure | Thompson (1998) | The impact of the demographics of aging and the edentulous condition on dental care services. |
| wrong population | Schembri (2001) | The implications of visual impairment in an elderly population in recognizing oral disease and maintaining oral health. |
| wrong outcome or exposure | Kamińska-Pikiewicz (2015) | The incidence of oral dryness in people over 65 years living in Lublin |
| wrong population | Vano (2015) | The influence of educational level and oral hygiene behaviours on DMFT index and CPITN index in an adult Italian population: an epidemiological study |
| wrong population | Pang (2022) | The mediating effect of social support on the association between socioeconomic status and self-reported oral health status among the migrant elderly following children in Weifang, China: a cross-sectional study. |
| wrong outcome or exposure | do Amaral (2023) | The mediating role of social capital in socioeconomic inequalities of oral health behaviours among Brazilian older adults. |
| wrong population | Abbass (2020) | The occurrence of periodontal diseases and its correlation with different risk factors among a convenient sample of adult Egyptian population: A cross-sectional study |
| cannot retrieve full text | Cautley (1992) | The oral health and attitudes to dental treatment of a dentate elderly population in Mosgiel, Dunedin. |
| wrong outcome or exposure | Santucci (2015) | The oral health of institutionalized older adults in Malta. |
| wrong outcome or exposure | Bergman (1991) | The oral health of the elderly in Melbourne. |
| wrong outcome or exposure | Riggs (2020) | The oral health quality of life for seniors in residential facilities who have direct access to care as compared to those without access. |
| cannot retrieve full text | Slade (1990) | The oral health status and treatment needs of adults aged 65+ living independently in Ottawa-Carleton |
| wrong population | Pengpid (2018) | The prevalence of edentulism and their related factors in Indonesia, 2014/15. |
| wrong population | Svatun (1974) | The provision of dental care for institutionalized mentally subnormal persons in Norway |
| wrong population | de Andrade (2011) | The relationship between nutrient intake, dental status and family cohesion among older Brazilians. |
| wrong population | Taraghi (2017) | The relationship between oral health and cognitive status of the elderly |
| wrong outcome or exposure | Masood (2017) | The relationship between oral health and oral health related quality of life among elderly people in United Kingdom. |
| wrong publication type | Elias (1998) | The relationship between satisfaction with mouth and number and position of teeth |
| wrong population | Sabbah (2011) | The relationship between social network, social support and periodontal disease among older Americans. |
| wrong outcome or exposure | Makhija (2006) | The relationship between sociodemographic factors and oral health-related quality of life in dentate and edentulous community-dwelling older adults. |
| wrong outcome or exposure | Doi (2023) | The relationship between subjective oral frailty and adverse health outcomes or medical and dental expenditures in the latter-stage older adult: A 6-year longitudinal study |
| wrong outcome or exposure | Hugoson (2002) | The relationship of some negative events and psychological factors to periodontal disease in an adult Swedish population 50 to 80 years of age. |
| cannot retrieve full text | Borisova (2000) | The risk factors and incidence of tooth loss in middle-aged and elderly persons |
| wrong publication type | Jablonski (2005) | The role of biobehavioral, environmental, and social forces on oral health disparities in frail and functionally dependent nursing home elders. |
| wrong population | Celeste (2013) | The role of potential mediators in racial inequalities in tooth loss: the Pró-Saúde study |
| wrong population | Jönsson (2020) | The role of psychosocial factors and treatment need in dental service use and oral health among adults in Norway |
| wrong outcome or exposure | Chung (2011) | The strength of age effect on tooth loss and periodontal condition in Korean elderly |
| wrong publication type | Actrn (2018) | Think Dental, Be Active!: a randomised control trial using psycho-education, physical activity and oral health interventions in older adults aged >50 years old, residing in Royal Freemason Benevolent Institute residential aged care facilities |
| wrong population | Drake (1995) | Three-year tooth loss among black and white older adults in North Carolina. |
| wrong publication type | van der Wall (2016) | Tooth brushing for a longer and healthier life |
| wrong population | Ibiyemi (2017) | Tooth loss among the elders in an inner city area of Ibadan, Nigeria. |
| wrong population | Dias (2019) | Tooth loss and associated factors in the elderly in Cruz Alta, Brazil: a crosssectional study. |
| wrong population | Susin (2005) | Tooth loss and associated risk indicators in an adult urban population from south Brazil. |
| wrong outcome or exposure | Northridge (2012) | Tooth loss and dental caries in community-dwelling older adults in northern Manhattan. |
| wrong outcome or exposure | Žmavc (2024) | Tooth Loss and Systemic Diseases in the Slovenian Elderly Population: A Cross-Sectional Study of the Associaton Between Oral and Systemic Health. |
| wrong outcome or exposure | Kato (2019) | Tooth loss-associated cognitive impairment in the elderly: A community-based study in Japan |
| wrong outcome or exposure | Peres (2015) | Tooth loss is associated with severe cognitive impairment among older people: findings from a population-based study in Brazil. |
| wrong population | Pallegedara (2005) | Tooth loss, the wearing of dentures and associated factors in Sri Lankan older individuals. |
| wrong population | Gilbert (1999) | Tooth-specific and person-level predictors of 24-month tooth loss among older adults. |
| wrong outcome or exposure | Ji (2024) | Trajectories of cognitive function and frailty in older adults in China: a longitudinal study |
| wrong outcome or exposure | Hybels (2016) | Trajectories of depressive symptoms and oral health outcomes in a community sample of older adults |
| wrong population | Hybels (2016) | Trends in decayed teeth among middle-aged and older adults in the United States: socioeconomic disparities persist over time. |
| wrong population | Andrade (2018) | Trends in socioeconomic inequalities in the prevalence of functional dentition among older people in Brazil. |
| wrong population | Matos (2007) | Trends in the use of dental services by elderly Brazilians and related socio-demographic factors based on the National Household Survey (1998 and 2003). |
| wrong population | Sevik (2025) | Understanding poor oral health among older adults in Türkiye: socioeconomic and healthcare access challenges. |
| wrong publication type | Ranjith (2024) | Understanding rapid oral health deterioration and its associated factors among older adults: A scoping review |
| wrong outcome or exposure | Park (2024) | Unmet Dental Care Needs of The Low-Income Elderly in South Korea: Applying the Andersen behavioural model. |
| wrong population | Araújo (2009) | Use of dental services and associated factors: a population-based study in southern Brazil. |
| cannot retrieve full text | Westaway (1999) | Utilisation of oral health services, oral health needs and oral health status in a peri-urban informal settlement |
| wrong publication type | Palmqvist (1989) | Utilization of dental services in an elderly population. |
| wrong outcome or exposure | Osterberg (1998) | Utilization of dental services in relation to socioeconomic and health factors in the middle-aged and elderly Swedish population. |
| wrong population | Nascimento (2024) | Validity of self-reported oral conditions among Brazilian older women: Do socio-economic factors matter? |
| wrong population | Brennan (1999) | Variation in dental service provision among adult migrant public-funded patients |
| wrong population | Slade (1996) | Variations in the social impact of oral conditions among older adults in South Australia, Ontario, and North Carolina. |
| wrong outcome or exposure | Tian (2025) | Veteran Status and Oral Health Indicators in Older Men: An Analysis Using the 2016 and 2018 Behavioral Risk Factor Surveillance System. |
| wrong population | De Marchi (2012) | Vulnerability and the psychosocial aspects of tooth loss in old age: a Southern Brazilian study. |
| wrong population | do Amaral (2023) | Wealth index association with self-reported oral health between white and non-white older Brazilians. |
| cannot retrieve full text | Tebbutt (2023) | What factors affect the ability of refugees to access dental care services? |
| wrong population | Ito (2020) | Wider Dental Care Coverage Associated with Lower Oral Health Inequalities: A Comparison Study between Japan and England. |
| wrong outcome or exposure | Avlund (2003) | Social relations as determinants of oral health among persons over the age of 80 years. |
| wrong outcome or exposure | Hoshi-Harada (2023) | Difference of income inequalities of denture use by co-payment rates: A JAGES cross-sectional study. |
| wrong outcome or exposure | Islas-Granillo (2017) | Dental prosthetic treatment needs in mexican elders: Influence of socioeconomic position |
| wrong outcome or exposure | Iwasaki (2025) | Dental visit avoidance during the COVID-19 pandemic is associated with oral frailty in Japanese community-dwelling older adults |
| wrong outcome or exposure | Mollaoglu (2005) | The effect of dental profile on daily functions of the elderly. |
| wrong outcome or exposure | Naka (2012) | Assessing oral health promotion determinants in active Greek elderly. |
| wrong outcome or exposure | Takahashi (2023) | Social Isolation/Loneliness and Tooth Loss in Community-Dwelling Older Adults: The Sukagawa Study. |
| Reporting same data from another included article (double reporting) | Yen (2015) | Impact of removable dentures on oral health-related quality of life among elderly adults in Taiwan. |
| wrong outcome or exposure | Gaszynska (2014) | Oral health status, dental treatment needs, and barriers to dental care of elderly care home residents in Lodz, Poland. |
| wrong outcome or exposure | Hu (2024) | An analysis of influencing factors of oral frailty in the elderly in the community. |
| wrong outcome or exposure | Lee (2013) | Cognitive function and oral health-related quality of life in older adults. |
| wrong outcome or exposure | Lexomboon (2012) | Chewing ability and tooth loss: Association with cognitive impairment in an elderly population study |
| wrong outcome or exposure | Ward (2020) | Dental attendance, restoration and extractions in adults with intellectual disabilities compared with the general population: a record linkage study |
| wrong population | Hanson (1994) | Social network, social support and dental status in elderly Swedish men. |
| wrong outcome or exposure | Smith (2020) | Barriers and enablers for dental care among dentate home-based older New Zealanders who receive living support. |
| wrong outcome or exposure | Borreani (2010) | Informing the debate on oral health care for older people: a qualitative study of older people's views on oral health and oral health care. |
| wrong outcome or exposure | Derblom (2017) | Older people's description of factors that facilitate and impede regular dental care - a qualitative interview study. |
| wrong outcome or exposure | McKenzie-Green (2009) | Older peoples' perceptions of oral health: 'it's just not that simple'. |
| wrong outcome or exposure | Gibson (2019) | Oral care as a life course project: A qualitative grounded theory study. |
| wrong outcome or exposure | Sussex (2010) | Understanding the 'epidemic' of complete tooth loss among older New Zealanders |
| wrong outcome or exposure | Takeda (2025) | Relationship Between Oral Function and Social Participation Among Community-Dwelling Older Adults: An Observational Cross-Sectional Study |
