# Supplementary material for: Association of Socioeconomic Factors With Oral Health in Older Adults: A Systematic Review and Meta‐Analysis
Source: Gerodontology. 2026 May 15;43(3):456–74. doi: 10.1111/ger.70079 (PMC13498931; doi:10.1111/ger.70079)
Supplement: Supplementary file 2 [file GER-43-456-s002.docx]

Appendix S3. Summary of findings on the association of socioeconomic factors with oral health.

**Table 1:** Summary of findings in quantitative studies on the association of socioeconomic factors with oral health

| **Exposures** | **Domains** | **Reference (n)** | **Strength of association** |
| --- | --- | --- | --- |
| SEP (occupation/ income/multi-aspect) | Dentition status | 30 studies  24 (157)  25 (411)  26 (168,122)  29 (1,654)  31 (11,476)  34 (335)  35 (1,722)  36 (Not mentioned)  37 (Not mentioned)  39 (463)  40 (769)  43 (3,600)  44 (760)  48 (322)  49 (712)  55 (176)  58 (179)^  60 (3,054)  62 (945)  65 (4,268)  66 (1,612)  71 (1,616)  72 (237)  73 (135,192)  79 (357)  81 (346)  83 (524)  85 (739)  86 (5,513)  90 (7,796) | **Meta-analysis (based on unadjusted data)**  Low SEP vs. Mean number of missing teeth^49, 72, 81, 83^: MD 2.11, 95%CI 0.73 to 3.50 (Figure 2a, S1)  Low SEP vs. Edentulism^26, 34, 35, 43, 60, 66, 71, 73, 81, 90^: OR 1.53, 95%CI 1.18 to 1.97 (Figure 3a, S3)  Low SEP vs. Lacking functional dentition^29, 40, 48, 60, 66, 71, 90^: OR 1.59, 95%CI 1.15 to 2.19 (Figure 4a, S4)  **Unpooled analyses (adjusted) (n = 11)**  Blue-collar occupations (reference higher positions) vs. Edentulism OR 5.6, 95%CI 0.6 to 56.6; White-collar occupations (reference higher positions) vs. Edentulism OR 4.3, 95%CI 0.4 to 43.8^24^  Low income vs. No or few teeth OR 2.0, 95%CI 1.3 to 3.1^25^  Second Q of neighborhood SEP deprivation (reference first Q, least deprived) vs. Edentulism OR 1.29, 95%CI 0.88 to 1.91; Third Q (reference first Q) vs. Edentulism OR 1.17, 95%CI 0.76 to 1.81; Fourth Q (reference first Q) vs. Edentulism OR 1.87, 95%CI 1.21 to 2.87; Fifth Q (reference first Q) vs. Edentulism OR 2.22, 95%CI 1.41 to 3.51; *P* for trend < 0.001^35^  [8-year cohort data] Second Q of neighborhood SEP deprivation (reference first Q, least deprived) vs. Tooth loss OR 1.16, 95%CI 0.77 to 1.75; Third Q (reference first Q) vs. Tooth loss OR 1.74, 95%CI 1.06 to 2.83; Fourth Q (reference first Q) vs. Tooth loss OR 1.51, 95%CI 0.83 to 2.74; Fifth Q (reference first Q) vs. Tooth loss OR 2.32, 95%CI 1.09 - 4.89; *P* for trend 0.01^35^  Skilled and unskilled workers, disability pensioners, housewives vs. Missing tooth surfaces B 19.3, 95%CI 4.4 to 34.2, *P* < 0.05; Low income at 85 years vs. Missing tooth surfaces B 4.8, 95%CI -13.0 to 22.6, *NS*^55^  Income vs. Edentulism *NS*^60^  Bad economic situations vs. Edentulism B 0.35, SE 0.12, *P* < 0.01^62^  [8-year cohort data] Enough financial sufficiency vs. Number of remaining teeth B -0.02, 95%CI -0.07 to 0.04, *NS*^65^  Financial situation, social class vs. Number of teeth *NS*; Middle social class (reference high social class) vs. Becoming edentulous before 40 years OR 7.7, 95%CI 1.0 to 59.2, *P* = 0.050; Low social class (reference high social class) vs. Becoming edentulous before 40 years OR 11.6, 95%CI 1.5 to 87.4, *P* = 0.017; Social class vs. Becoming edentulous between 40 - 70 years *NS*^79^  Income vs. Oral health (subjective sense of health, number of remaining teeth, and oral function) B 0.10, *P* < 0.05^85^  Second, third, fourth Q of household income (reference first Q, least income) vs. Number of teeth < 20 *NS* (males, females); Fifth Q (reference first Q) vs. Number of teeth < 20: *P* = 0.045 (males), *NS* (females)^86^  Economic status vs. Tooth loss B = -0.057, 95%CI -0.094 to -0.020, *P* < 0.01; Professional occupation vs. Tooth loss B = -0.245, 95%CI -0.307 to -0.181, *P* < 0.01^90^  **Unpooled analyses (unadjusted) (n = 12)**  Blue-collar occupations (reference higher positions) vs. Edentulism OR 5.3, 95%CI 0.7 to 43.3; White-collar occupations (reference higher positions) vs. Edentulism OR 3.7, 95%CI 0.4 to 31.8^24^  Low income vs. No or few teeth OR 2.0, 95%CI 1.3 to 3.1^25^  Poverty status (poor, near poor, nonpoor) vs. functional dentition: Edentulism declined and functional dentition (>20 teeth) increased across all groups, with the greatest improvements among the poor. Edentulism fell from 47.1% to 36.4% (poor), 39.5% to 30.1% (near poor), and 18.0% to 14.9% (nonpoor). Functional dentition rose from 16.0% to 21.3%, 25.4% to 34.7%, and 44.7% to 58.6%, respectively. Mean teeth count also increased modestly in all groups^31^  Income vs. Edentulism: The edentulism incidence was highest in the first quintile (the lowest income group)^36^  Income vs. Functional dentition: Mean number of teeth *P* < 0.05; Edentulism *P* < 0.05^37^  Dissatisfied economic status vs. No posterior occlusal support OR 1.53, 95%CI 0.94 to 2.50^39^  Dissatisfied economic status vs. Low number of teeth (0 - 9 teeth) OR 1.17, 95%CI 0.80 to 1.69^44^  Skilled and unskilled workers, disability pensioners, housewives vs. Missing tooth surfaces Bivariate mean 24.7, 95%CI 11.6 to 37.8, *P* < 0.05; Low income at 85 years vs. Missing tooth surfaces Bivariate mean 16.5, 95%CI 4.0 to 29.1, *P* < 0.05^55^  Low socioeconomic status vs. Functional dentition: Mean number of missing teeth MD 3.6, 95%CI -0.82 to 8.10; Edentulism OR 2.58, 95%CI 1.23 to 5.41^58^  Income vs. Oral health (subjective sense of health, number of remaining teeth, and oral function) r = 0.161, *P* < 0.001^85^  Second Q of household income (reference first Q, least income) vs. Number of teeth < 20 *NS* (males, females); Third Q (reference first Q) vs. Number of teeth < 20: *P* = 0.005 (males), *NS* (females); Fourth Q (reference first Q) vs. Number of teeth < 20: *P* < 0.001 (males), *NS* (females) Fifth Q (reference first Q) vs. Number of teeth < 20: *P* < 0.001 (males), *P* = 0.006 (females)^86^  Non-professional vs. Edentulism OR 1.95, 95%CI 1.63 to 2.32; Number of teeth ≤ 20 OR 2.05, 95%CI 1.79 to 2.36^90^ |
| Education | Dentition status | 25 studies  23 (21,736)  24 (157)  25 (411)  30 (5,403)  34 (335)  39 (463)  40 (782)  43 (3,600)  44 (760)  46 (139)  48 (322)  51 (241)  53 (834)  55 (176)  60 (3,054)  63 (238)  64 (1,210)^  65( 4,268)  69 (257)^  75 (170)  77 (523)  79 (357)  85 (739)  86 (2,993)  90 (7,796) | **Meta-analysis (based on unadjusted data)**  Low education level vs. Mean number of missing teeth^46, 63, 75^ : MD 2.61, 95%CI 1.15 to 4.06 (Figure 2b, S2)  Low education level vs. Edentulism^30, 34, 43, 51, 53, 60, 63, 77, 90^ : OR 1.85, 95%CI 1.45 to 2.36 (Figure 3b, S7)  Low education level vs. Number of teeth < 20^23, 30, 40, 48, 53, 60, 75, 77, 90^ : OR 2.18, 95%CI 1.58 to 3.00 (Figure 4b, S8)  **Unpooled analyses (adjusted) (n = 11)**  Education 10 - 12 years (reference ≤ 9 years) vs. Remaining teeth > 20 OR 0.99, 95%CI 0.91 to 1.08; Education 13 - 15 years (reference ≤ 9 years) vs. Remaining teeth > 20 OR 1.04, 95%CI 0.94 to 1.15; Education ≥ 16 years (reference ≤ 9 years) vs. Remaining teeth > 20 OR 1.12, 95%CI 0.97 to 1.30^23^  Education 2 - 7 years (reference ≥ 11 years) vs. Edentulism OR 1.7, 95%CI 0.5 to 5.8; Education 8 - 10 years (reference ≥ 11 years) vs. Edentulism OR 0.2, 95%CI 0.0 to 1.1^24^  Low education level (< 3 years) vs. No or few teeth OR 2.3, 95%CI 1.5 to 3.6^25^  Low education level (vocational education < 3 years) vs. Missing tooth surfaces B 11.9, 95%CI -2.7 to 27.6, *NS*^55^  Education level vs. Edentulism *NS*^60^  [7-year cohort data] Education 1 - 6 years (reference 0 year of schooling) vs. Number of remaining teeth B 0.08, 95%CI 0.02 to 0.13, P < 0.01; Education > 6 years (reference 0 year of schooling) vs. Number of remaining teeth B 0.18, 95%CI 0.11 to 0.25, P < 0.001^65^  Low education level (secondary school or less) vs. Number of missing teeth B 0.10, IRR 10.65 (0.27 - 22.11), *P* = 0.044^69^  High education level vs. Number of teeth (between 0 teeth and ≥ 20 teeth) OR 5.8, 95%CI 2.3 to 14.6, *P* < 0.001; High education level vs. Number of teeth (between 1 - 10 teeth and ≥ 20 teeth) *NS*; Elementary school (reference higher education) vs. Becoming edentulous before 40 years *NS*; Elementary school (reference higher education) vs. Becoming edentulous between 40 - 70 years OR 4.0, 95%CI 1.8 to 8.7, *P* = 0.001^79^  Education vs. Oral health (subjective sense of health, number of remaining teeth, and oral function) B 0.135, *P* < 0.01^85^  Having education vs. Number of teeth < 20: OR 1.16, 95%CI 0.77 to 1.73 (males); OR 0.94, 95%CI 0.65 to 1.38 (females)^86^  Education vs. Tooth loss B = -0.121, 95%CI -0.168 to -0.074, *P* < 0.01^90^  **Unpooled analyses (unadjusted) (n = 8)**  Education 2 - 7 years (reference ≥ 11 years) vs. Edentulism OR 1.6, 95%CI 0.6 to 4.1; Education 8 - 10 years (reference ≥ 11 years) vs. Edentulism OR 0.5, 95%CI 0.2 to 1.6^24^  Low education level (< 3 years) vs. No or few teeth OR 2.3, 95%CI 1.5 to 3.6^25^  Low education level (junior high school) vs. No posterior occlusal support OR 1.58, 95%CI 1.04 to 2.38^39^  Low education level ( ≤ 9 years) vs. Low number of teeth (0 - 9 teeth) OR 1.59, 95%CI 1.13 to 2.22^44^  Low education level (vocational education < 3 years) vs. Missing tooth surfaces Bivariate mean 20.0, 95%CI 7.6 to 32.4^55^  Low education level (high school or less) vs. Mean number of missing teeth MD 3.05, 95%CI 2.04 to 4.06^64^  Low education level (secondary school or less) vs. Mean number of missing teeth MD 2.78, 95%CI 0.13 to 5.43^69^  Education vs. Oral health (subjective sense of health, number of remaining teeth, and oral function) r = 0.192, *P* < 0.001^85^ |
| SEP (occupation/ income/multi-aspect) | Oral conditions: dental caries | 8 studies  24 (157)  32 (864)  37 (Not mentioned)  55 (176)  58 (90)^  72 (237)  81 (135)  83 (524) | **Meta-analysis (based on unadjusted data)**  Low SEP vs. Coronal caries^72, 81, 83^: MD 0.45, 95%CI 0.15 to 0.75 (Figure S11, S12)  **Unpooled analyses (adjusted) (n = 1)**  Blue-collar occupations (reference higher positions) vs. Coronal caries OR 5.9, 95%CI 1.3 to 26.5; White-collar occupations (reference higher positions) vs. Coronal caries OR 5.4, 95%CI 1.3 to 22.6^24^  **Unpooled analyses (unadjusted) (n = 7)**  Blue-collar occupations (reference highest) vs. Coronal caries OR 3.4, 95%CI 1.0 to 11.8; White-collar occupations (reference highest) vs. Coronal caries OR 4.2, 95%CI 1.2 to 14.9; Blue-collar occupations (reference highest) vs. Root caries OR 1.2, 95%CI 0.4 to 3.6; White-collar occupations (reference highest) vs. Root caries OR 0.8, 95%CI 0.3 to 2.6^24^  Limit financial resources for dental care vs. Having caries lesion OR 1.31, 95%CI 0.82 to 2.10^32^  Income vs. Dental caries parameters: Coronal caries *P* < 0.05; Root caries *NS*^37^  Skilled and unskilled workers vs. Decayed tooth surfaces Bivariate mean 2.0, 95%CI -3.2 to 7.2, *NS*; Low income at 85 years vs. Decayed tooth surfaces Bivariate mean -1.3, 95%CI -6.0 to 3.4, *NS*^55^  SEP vs. Mean number of coronal caries (SD): High 1.0 (1.4), Medium 1.4 (2.0), Low 1.3 (1.7), *NS*; SEP vs. Mean number of root caries: High 0.6 (0.9), Medium 1.0 (1.4), Low 0.4 (0.7), *NS*^58^  Low income vs. Number of decayed root surfaces MD 0.84, 95%CI -0.15 to 1.83; Low income vs. Number of decayed and/or filled root surface MD -10.63, 95%CI -15.13 to -6.13^83^ |
| Education | Oral conditions: dental caries | 5 studies  24 (157)  32 (835)  55 (176)  63 (155)  74 (71) | **Unpooled analyses (adjusted) (n = 2)**  Education 2 - 7 years (reference ≥ 11 years) vs. Coronal caries OR 0.6, 95%CI 0.2 to 1.8; Education 8 - 10 years (reference ≥ 11 years) vs. Coronal caries OR 0.8, 95%CI 0.3 to 2.2^24^  [5-year cohort data] Education level vs. Root caries index increment B 0.017, 95%CI -0.034 to 0.069, *P* = 0.496^74^  **Unpooled analyses (unadjusted) (n = 5)**  Education 2 - 7 years (reference ≥ 11 years) vs. Coronal caries OR 0.6, 95%CI 0.3 to 1.5; Education 8 - 10 years (reference ≥ 11 years) vs. Coronal caries OR 1.0, 95%CI 0.4 to 2.3; Education 2 - 7 years (reference ≥ 11 years) vs. Root caries OR 1.1, 95%CI 0.5 to 2.7; Education 8 - 10 years (reference ≥ 11 years) vs. Root caries OR 0.7, 95%CI 0.3 to 1.7^24^  Low education level vs. Having caries lesion OR 1.48, 95%CI 0.93 to 2.36^32^  Low education level (vocational education level < 3 years) vs. Decayed tooth surfaces Bivariate mean 0.8, 95%CI -3.4 to 5.0, *NS*^55^  Low education level (elementary school or less) vs. Decayed surface MD 0.2, *P* = 0.04^63^  Low education level (< 7 years) vs. Dental caries parameters: Decayed root surface MD -0.6, 95%CI -1.2 to 0.1; Root caries index MD -0.02, 95%CI -0.10 to 0.06^74^ |
| SEP (occupation/ income/multi-aspect) | Oral conditions: periodontal disease | 6 studies  35 (1,722)  37 (Not mentioned)  41 (101)  66 (1,215)  70 (245)  83 (296) | **Unpooled analyses (adjusted) (n = 2)**  Second Q of neighborhood SEP deprivation (reference first Q, least deprived) vs. 20% sites affected by periodontal pockets > 3.5 mm OR 1.48, 95%CI 1.03 to 2.13; Third Q (reference first Q) vs. 20% sites affected by periodontal pockets > 3.5 mm OR 1.60, 95%CI 1.07 to 2.39; Fourth Q (reference first Q) vs. 20% sites affected by periodontal pockets > 3.5 mm OR 1.39, 95%CI 0.88 to 2.21; Fifth Q (reference first Q) vs. 20% sites affected by periodontal pockets > 3.5 mm OR 2.82, 95%CI 1.72 to 4.64; *P* for trend < 0.001^35^  Higher social class vs. Serious periodontitis OR 1.68, 95%CI 0.62 to 4.56^41^  **Unpooled analyses (unadjusted) (n = 5)**  Income vs. Having periodontal disease *P* < 0.05^37^  Higher social class vs. Serious periodontitis OR 2.50, 95%CI 1.04 to 6.00^41^  Manual classes vs. Periodontal parameters: Periodontal disease based on > 20% of sites with loss of attachment > 5.5 mm OR 1.45, 95%CI 1.12 to 1.89; Periodontal pocket depth > 20% of sites with > 3.5 mm pocket depth OR 1.26, 95%CI 0.98 to 1.61; Gingival bleeding OR 0.87, 95%CI 0.63 to 1.19^66^  Low annual household income vs. High scores periodontal inflamed surface area OR 0.35, 95%CI 0.12 to 1.02^70^  Low income vs. Periodontal parameters: Percentage of tooth sites with pocket depth ≥ 3 mm MD 5.80, 95%CI 4.47 to 7.13; Clinical attachment loss ≥ 5 mm MD -0.70, 95%CI -2.38 to 0.98; Gingival index ≥ 2 MD 4.97, 95%CI 3.35 to 6.59^83^ |
| Education | Oral conditions: periodontal disease | 8 studies  41 (101)  63 (155)  64 (1,210)^  67 (1,241)  70 (245)  74 (71)  75 (170)  76 (154) | **Unpooled analyses (adjusted) (n = 1)**  High education vs. Serious periodontitis OR 1.35, 95%CI 0.54 to 3.40^41^  **Unpooled analyses (unadjusted) (n = 8)**  High education vs. Serious periodontitis OR 2.01, 95%CI 0.86 to 4.68^41^  Low education level (elementary school or less) vs. Periodontal parameters: Probing attachment level MD 0.29, 95%CI -0.21 to 0.80; Periodontal treatment needs *NS*^63^  Low education level (high school or less) vs. Periodontal parameters: Having sub-gingival calculus OR 1.07, 95%CI 0.78 to 1.46; Having gingival bleeding OR 1.40, 95%CI 1.03 to 1.90; Prevalence of CAL ≥ 5 mm OR 0.87, 95%CI 0.59 to 1.28; CAL MD 0.11, 95%CI -0.03 to 0.23; Percent of sites with CAL ≥ 5 mm MD 2.77, 95%CI 0.23 to 5.31; Prevalence of PD ≥ 6 mm OR 0.98, 95%CI 0.71 to 1.36; Pocket depth MD 0.02 , 95%CI -0.05 to 0.09; Percent of sites with PD ≥ 6 mm MD 0.50, 95%CI -0.15 to 1.15; Severe periodontitis (European) OR 1.42, 95%CI 1.04 to 1.93; Severe periodontitis (CDC/AAP) OR 1.23, 95%CI 0.88 to 1.73^64^  Low education level (primary school or less) vs. Oral health using Oral Health Assessment Tool MD 0.25, 95%CI 0.01 to 0.49^67^  Low education level (< 10 years) vs. High scores periodontal inflamed surface area OR 0.90, 95%CI 0.50 to 1.63^70^  Low education level (< 7 years) vs. Exposed root surfaces OR -16.65, 95%CI -24.31 to -8.99^74^  Low education level (< 7 years) vs. Periodontal parameters: Having periodontal disease OR 1.3, 95%CI 0.7 to 2.5; Number of sextants with periodontal pockets ≥ 4 mm MD -0.3, 95%CI -0.8 to 0.2; Calculus MD 0.0, 95%CI -0.3 to 0.3; 1 -3 mm vertical bone loss MD -0.8, 95%CI -1.7 to 0.1; 4 -6 mm vertical bone loss MD 0.0, 95%CI -0.3 to 0.3; > 6 mm vertical bone loss MD 0.0, 95%CI -0.2 to 0.2; Furcation lesion MD -0.7, 95%CI -1.3 to -0.1^75^  Low education level (< 7 years) vs. Periodontal pockets ≥ 4 mm RR 0.9, 95%CI 0.7 to 1.1^76^ |
| SEP (occupation/ income/multi-aspect) | Oral conditions: dry mouth | 1 study  35 (2,137) | **Unpooled analyses (adjusted) (n = 1)**  Second Q of neighborhood SEP deprivation (reference first Q, least deprived) vs. Xerostomia OR 1.35, 95%CI 1.05 to 1.73; Third Q (reference first Q) vs. Xerostomia OR 1.04, 95%CI 0.79 to 1.38; Fourth Q (reference first Q) vs. OR 1.35, 95%CI 1.00 to 1.83; Fifth Q (reference first Q) vs. Xerostomia OR 1.51, 95%CI 1.08 to 2.09; *P* for trend < 0.03^35^  [8-year cohort data] Second Q of neighborhood SEP deprivation (reference first Q, least deprived) vs. Xerostomia B -0.04, 95%CI -0.47 to 0.38, *NS*; Third Q (reference first Q) vs. Xerostomia B 0.19, 95%CI -0.32 to 0.69, *NS*; Fourth Q (reference first Q) vs. Xerostomia B 0.37, 95%CI -0.26 to 0.99, *NS*; Fifth Q (reference first Q) vs. Xerostomia B 0.31, 95%CI -0.41 to 1.05, *NS*; *P* for trend 0.16^35^ |
| Education | Oral conditions: dry mouth | 1 study  47 (139) | **Unpooled analyses (unadjusted) (n = 1)**  Education level vs. saliva flow (amount): *P* = 0.641^47^ |
| SEP (occupation/ income/multi-aspect) | Oral function | 2 studies  25 (411)  57 (166)^ | **Unpooled analyses (adjusted) (n = 1)**  Sufficient economic status (reference affluent) vs. Poor chewing efficiency OR 8.94, 95%CI 3.00 to 26.65^57^  **Unpooled analyses (unadjusted) (n = 1)**  Low income vs. Difficulty chewing OR 1.0, 95%CI 0.7 to 1.6^25^ |
| Education | Oral function | 3 studies  25 (411)  51 (241)  57 (166)^ | **Unpooled analyses (adjusted) (n =1)**  Low education level (< 3 years) vs. Difficulty chewing OR 1.7, 95%CI 1.1 to 2.5^25^  **Unpooled analyses (unadjusted) (n = 3)**  Low education level (< 3 years) vs. Difficulty chewing OR 1.5, 95%CI 1.0 to 2.3^25^  Low education level (< Primary school) vs. Poor chewing ability OR 2.91, 95%CI 1.63 to 5.21^51^  Junior high school and under (reference college and above) vs. Poor chewing efficiency OR 2.01, 95%CI 0.82 to 4.93; Senior high school (reference college and above) vs. Poor chewing efficiency OR 2.42, 95%CI 0.99 to 5.88^57^ |
| SEP (occupation/ income/multi-aspect) | Oral health behaviours | 8 studies  24 (157)  25 (411)  27 (1,751)  33 (269)  61 (126)  73 (135,192)  81 (346)  88 (344)^ | **Meta-analysis (based on unadjusted data)**  Low SEP vs. No regular use of dental services^33, 61, 73, 81^ : OR 2.42, 95%CI 1.60 to 3.66 (Figure S13, S14)  Low SEP vs. Low frequency of toothbrushing^33, 61, 81^: OR 1.87, 95%CI 0.44 to 7.93 (Figure S16)  **Unpooled analyses (adjusted) (n = 5)**  Blue-collar occupations (reference higher positions) vs. No regular use of dental services OR 2.0, 95%CI 0.3 to 12.3; White-collar occupations (reference higher positions) vs. No regular use of dental services OR 1.1, 95%CI 0.2 to 5.3^24^  Low income vs. No regular use of dental services OR 1.6, 95%CI 1.6 to 4.2^25^  Higher income vs. Dental visitation: *NS* (dentate); OR 2.00, 95%CI 1.25 to 3.17 (edentulous); Higher satisfaction with income vs. Dental visitation *NS* (dentate, edentulous); Being employed vs. Dental visitation: *NS* (dentate, edentulous)^27^  SEP vs. Dental service use frequency OR 1.74, 95%CI 0.97 to 3.14^61^  High income (retirement pension) vs. Dental care attendance OR 1.82, 95%CI 1.07 to 3.10^88^  **Unpooled analyses (unadjusted) (n = 4)**  Blue-collar occupations (reference higher positions) vs. No regular use of dental services OR 1.7, 95%CI 0.4 to 6.5; White-collar occupations (reference higher positions) vs. No regular use of dental services OR 0.8, 95%CI 0.2 to 3.7^24^  Low income vs. No regular use of dental services OR 2.5, 95%CI 1.5 to 4.0^25^  Higher income vs. Dental visitation *P* < 0.05 (dentate, edentulous); Higher satisfaction with income vs. Dental visitation: *P* < 0.05 (dentate, edentulous); Being employed vs. Dental visitation: *P* < 0.05 (dentate, edentulous)^27^  Low income vs. No regular use of dental service OR 1.87, 95%CI 1.16 to 3.02^88^ |
| Education | Oral health behaviours | 9 studies  24 (157)  25 (411)  27 (1,751)  53 (834)  54 (168)  59 (354)  68 (530)  78 (633)  88 (299)^ | **Meta-analysis (based on unadjusted data)**  Low education level vs. No regular use of dental services ^53, 68^ : OR 3.05, 95%CI 0.00 to 6745.81 (Figure S15)  **Unpooled analyses (adjusted) (n = 5)**  Education 2 - 7 years (reference ≥ 11 years) vs. No regular use of dental services OR 2.6, 95%CI 0.7 to 9.2; Education 8 - 10 years (reference ≥ 11 years) vs. No regular use of dental services OR 1.2, 95%CI 0.3 to 5.2^24^  Low education level (< 3 years) vs. No regular use of dental services OR 2.3, 95%CI 1.5 to 3.6^25^  Higher education vs. Dental visitation: OR 1.47, 95%CI 1.09 to 2.00 (dentate); OR 1.34, 95%CI 1.07 to 1.69 (edentulous)^27^  School education vs. Regular dental care OR 5.34, 95%CI 2.44 to 11.70^59^  Education vs. Number of dental visits B 0.18, SE = 0.062, *P* < 0.01^78^  **Unpooled analyses (unadjusted) (n = 5)**  Education 2 - 7 years (reference ≥ 11 years) vs. No regular use of dental services OR 1.8, 95%CI 0.7 to 4.7; Education 8 - 10 years (reference ≥ 11 years) vs. No regular use of dental services OR 1.1, 95%CI 0.4 to 3.1^24^  Low education level (< 3 years) vs. No regular use of dental services OR 2.3, 95%CI 1.4 to 3.6^25^  Higher education vs. Dental visitation: *P* < 0.05 (dentate); *NS* (edentulous)^27^  Low education level (< 7 years) vs. Dental behaviors: Good oral hygiene (plaque < 20%) OR 0.9, 95%CI 0.5 to 1.7; Toothbrushing at least twice a day OR 0.8, 95%CI 0.4 to 1.7; Toothpaste use at least twice a day OR 0.6, 95%CI 0.3 to 1.1^54^  Low education level (elementary or less) vs. No regular use of dental services OR 1.46, 95%CI 0.92 to 2.30^88^ |
| SEP (occupation/ income/multi-aspect) | OHRQoL | 8 studies  42 (332)^  50 (641)  52 (409)  56 (202)^  57 (166)^  80 (1,040)  84 (195)  87 (344)^ | **Meta-analysis (based on unadjusted data)**  Low SEP vs. Poor OHRQoL^52, 80^: SMD 0.17, 95%CI 0.07 to 0.28 (Figure S17)  **Unpooled analyses (adjusted) (n = 6)**  Can pay an expected dental bill without difficulty (reference cannot pay) vs. Poor OHRQoL using OHIP-14 OR 0.43, 95%CI 0.18 to 0.97; Able to pay with difficulty (reference cannot pay) vs. Poor OHRQoL using OHIP-14 OR 0.64, 95%CI 0.31 to 1.32^52^  High income vs. Poor OHRQoL using OHIP-14: B -1.125, SE = 0.737, OR 0.325, *P* = 0.127 (women); B 0.019, SE = 0.987, OR 1.019, *P* = 0.985 (men)^56^  Low economic status vs. Better OHRQoL using GOHAI OR 0.30, 95%CI 0.13 to 0.71^57^  State pension only vs. Better OHRQoL using GOHAI B -0.48, 95%CI -1.37 to 0.42, *P* = 0.293^80^  Clerical/skilled workers (reference service/labour) vs. Better OHRQoL using GOHAI B -0.01, 95%CI -3.40 to 3.93, *P* = 0.998; Managerial/professional (reference service/labour) vs. Better OHRQoL using GOHAI B 0.81, 95%CI -2.97 to 4.59, *P* = 0.672^84^  Income vs. Poor OHRQoL using OHIP-14 OR 6.17, 95%CI 0.46 to 8.25, *P* = 0.17^87^  **Unpooled analyses (unadjusted) (n = 3)**  Annual household income vs. Poor OHRQoL using OHIP-14 r = -0.03, *P* = 0.45^50^  Low economic status vs. Better OHRQoL using GOHAI OR 0.25, 95%CI 0.11 to 0.54^57^  Low income vs. Better OHRQoL using GOHAI MD -0.93, 95%CI -3.53 to 1.67^42^ |
| Education | OHRQoL | 12 studies  28 (647)  38 (158)  42 (332)^  45 (50)  52 (409)  56 (202)^  57 (166)^  80 (1,040)  82 (675)  84 (195)  87 (344)^  89 (295) | **Meta-analysis (based on unadjusted data)**  Low education level vs. Poor OHRQoL^28, 52, 80^ : SMD 0.21, 95%CI 0.11 to 0.30 (Figure S18, S19)  **Unpooled analyses (adjusted) (n = 11)**  1 - 6 years (reference no schooling) vs. Poor OHRQoL using OHIP-14 RR 1.26, 95%CI 0.86 to 1.84; 7 - 9 years (reference no schooling) vs. Poor OHRQoL using OHIP-14 RR 1.23, 95%CI 0.78 to 1.96; 10 - 12 years (reference no schooling) vs. Poor OHRQoL using OHIP-14 RR 1.29, 95%CI 0.80 to 2.10; > 13 years (reference no schooling) vs. Poor OHRQoL using OHIP-14 RR 0.88, 95%CI 0.56 to 1.39^28^  No graduation or in a school of the lowest education (reference high education or studies) vs. Poor OHRQoL using OHIP-14 B 1.39, 95%CI 0.21 to 2.57, *P* = 0.021; Middle school level (reference high education or studies) vs. Poor OHRQoL using OHIP-14 B 0.77, 95%CI -0.44 to 1.97 *P* = 0.21^38^  High school (reference less than high school) vs. Better OHRQoL using GOHAI B -1.90, 95%CI -3.64 to -0.16, β -0.12, *P* = 0.032; College/university (reference less than high school) vs. Better OHRQoL GOHAI B -3.22, 95%CI -5.32 to -1.12, β -0.17, *P* = 0.003^42^  0 - 6 years education attainment (reference ≥ 10 years) vs. Poor OHRQoL using OHIP-14 OR 2.02, 95%CI 0.83 to 4.88; 7 - 9 years education attainment (reference ≥ 10 years) vs. Poor OHRQoL using OHIP-14 OR 1.48, 95%CI 0.72 to 3.04^52^  High education level (> elementary school) vs. Poor OHRQoL using OHIP-14: B 0.824, SE = 0.754, OR 2.281, *P* = 0.274 (women); B -0.646, SE = 0.877, OR 0.524, *P* = 0.461 (men)^56^  Junior high school and under (reference college and above) vs. Better OHRQoL using GOHAI OR 0.70, 95%CI 0.28 to 1.75; Senior high school (reference college and above) vs. Better OHRQoL using GOHAI OR 0.43, 95%CI 0.17 to 1.13^57^  Education level vs. Better OHRQoL using GOHAI B 0.75, 95%CI 0.13 to 1.37, *P* = 0.017^80^  High education level (> middle school) vs. Poor OHRQoL using OHIP-14 OR 0.73, 95%CI 0.48 to 1.09^82^  High education level (> primary school) vs. Better OHRQoL using GOHAI B 1.54, 95%CI -1.60 to 4.68, *P* = 0.332^84^  Low education level vs. Poor OHRQoL using OHIP-14 OR 2.17, 95%CI 1.25 to 3.75, *P* = 0.006^87^  High school (reference no studies or primary school) vs. Poor OHRQoL using GOHAI OR 0.54, *P* = 0.21; Technical or university studies (reference no studies or primary school) vs. Poor OHRQoL using GOHAI OR 1.01, *P* = 0.98^89^  **Unpooled analyses (unadjusted) (n = 8)**  Education level vs. OHIP-14 *P* = 0.018^38^  High school (reference less than high school) vs. Better OHRQoL using GOHAI B -1.25, 95%CI -3.24 to 0.73, *P* = 0.215; College/university (reference less than high school) vs. Better OHRQoL using GOHAI B -1.06, 95%CI -3.41 to 1.30, *P* = 0.378^42^  Education level vs. OHIP-14 *P* = 0.522^45^  Junior high school and under (reference college and above) vs. Better OHRQoL using GOHAI OR 0.56, 95%CI 0.25 to 1.24; Senior high school (reference college and above) vs. Better OHRQoL using GOHAI OR 0.36, 95%CI 0.16 to 0.82^57^  Education level vs. Better OHRQoL using GOHAI B 1.18, 95%CI 0.61 to 1.74, *P* < 0.0001^80^  Low education level (middle school or less) vs. Poor OHRQoL using OHIP-14 OR 1.70, 95%CI 1.16 to 2.49^82^  Low education level (elementary or less) vs. Poor OHRQoL using OHIP-14 MD 1.8, 95%CI -0.56 to 4.16^87^  High school (reference no studies or primary school) vs. Poor OHRQoL using GOHAI OR 0.69, *P* = 0.25; Technical or university studies (reference no studies or primary school) vs. Poor OHRQoL using GOHAI OR 0.88, *P* = 0.74^89^ |

Note: Underline = Statistically significant; ^ = Reported using convenience samples; *NS* = Non-significant; SE = Standard error; CI = Confidence interval; OR = Odds Ratio; MD = Mean Difference; SMD = Standardized Mean Difference; IRR = Incidence Rate Ratio; B = B Coefficient; β = Beta; r = Pearson correlation coefficient; SEP = Socioeconomic position; OHRQoL = Oral Health–Related Quality of Life; OHIP-14 = Oral Health Impact Profile-14; GOHAI = Geriatric Oral Health Assessment Index; Q = Quintile.
