# Supplementary material for: Association of Socioeconomic Factors With Oral Health in Older Adults: A Systematic Review and Meta‐Analysis
Source: Gerodontology. 2026 May 15;43(3):456–74. doi: 10.1111/ger.70079 (PMC13498931; doi:10.1111/ger.70079)
Supplement: Supplementary file 3 [file GER-43-456-s003.docx]

Appendix S4. Supplementary forest and funnel plots of meta-analyses.

**Figure S1** Forest plot showing the population-stratified mean difference in the number of missing teeth by socioeconomic position

**Figure S2** Forest plot showing the population-stratified mean difference in the number of missing teeth by education

**Figure S3** Forest plot showing the population-stratified odds ratio in the prevalence of edentulism by socioeconomic position

**Figure S4** Forest plot showing the population-stratified odds ratio in the prevalence of lacking functional dentition by socioeconomic position

**Figure S5** Forest plot showing the odds ratio in the prevalence of edentulism by socioeconomic position (sensitivity analysis)

**Figure S6** Forest plot showing the odds ratio in the prevalence of lacking functional dentition by socioeconomic position (sensitivity analysis)

**Figure S7** Forest plot showing the population-stratified odds ratio in the prevalence of edentulism by education

**Figure S8** Forest plot showing the population-stratified odds ratio in the prevalence of lacking functional dentition by education

**Figure S9** Forest plot showing the odds ratio in the prevalence of edentulism by education levels (sensitivity analysis)

**Figure S10** Forest plot showing the odds ratio in the prevalence of lacking functional dentition by education levels (sensitivity analysis)

**Figure S11** Forest plot showing the mean difference in the number of dental/coronal caries teeth by socioeconomic position

**Figure S12** Forest plot showing the population-stratified mean difference in the number of dental/coronal caries teeth by socioeconomic position

**Figure S13** Forest plot showing the odds ratio in the irregular use of dental services by socioeconomic position

**Figure S14** Forest plot showing the population-stratified odds ratio in the irregular use of dental services by socioeconomic position

**Figure S15** Forest plot showing the odds ratio in the irregular use of dental services by education levels

**Figure S16** Forest plot showing the odds ratio of the low frequency of toothbrushing by socioeconomic position

**Figure S17** Forest plot showing the standardised mean difference in Oral Health-Related Quality of Life by socioeconomic position

**Figure S18** Forest plot showing the standardised mean difference in Oral Health-Related Quality of Life by education levels

**Figure S19** Forest plot showing the population-stratified standardised mean difference in Oral Health-Related Quality of Life by education levels

Figure S20 Funnel plot: the odds ratio in the prevalence of edentulism by socioeconomic position

Figure S21 Funnel plot: the odds ratio in the prevalence of edentulism by education levels

Figure S22 Funnel plot: the odds ratio in the prevalence of lacking functional dentition by socioeconomic position

Figure S23 Funnel plot: the odds ratio in the prevalence of lacking functional dentition by education levels
