# Supplementary material for: Association of Socioeconomic Factors With Oral Health in Older Adults: A Systematic Review and Meta‐Analysis
Source: Gerodontology. 2026 May 15;43(3):456–74. doi: 10.1111/ger.70079 (PMC13498931; doi:10.1111/ger.70079)
Supplement: Supplementary file 4 [file GER-43-456-s004.docx]

Appendix S2. Risk of bias and quality assessment of included studies.

Table 1 Quality assessment for cohort studies using the Newcastle Ottawa Scale (NOS)^21^ –

|  | Selection | | | | Comparability | Outcome | | |  |
| --- | --- | --- | --- | --- | --- | --- | --- | --- | --- |
| Author (Year) | Representativeness of the exposed cohort | Selection of the non-exposed cohort | Ascertainment of exposure | Demonstration that outcome of interest was not present at start of study | Comparability of cohorts on the basis of the design or analysis | Assessment of outcome | Was follow-up long enough for outcomes to occur | Adequacy of follow-up of cohorts | Quality |
| Ganbavale, S.G., Papachristou, E., Mathers, J.C. et al. (2024) ^35^ | * | * | * | - | ** | - | * | - | Fair |
| Qi, X., Pei, Y., Wang, K. et al. (2023) ^65^ | * | * | * | * | ** | - | * | - | Fair |
| Siukosaari, P., Ainamo, A., and Narhi, T.O. (2005) ^74^ | * | * | * | * | - | * | * | - | Poor |

Table 2 Risk of bias assessment for cross-sectional studies with association data using adapted NOS^22^ –

|  | Study sample selection | | Assessment of exposure and outcome | | Confounding factors | |  |
| --- | --- | --- | --- | --- | --- | --- | --- |
| Author (Year) | Representativeness of the study sample | Sample size | Assessment of the exposure(s) | Assessment of the outcome(s) | Adjustment for confounder(s) | Assessment of confounder(s) | Risk of bias |
|  |  |  |  |  |  |  |  |
| Aida, J., Kuriyama, S., Ohmori-Matsuda, K. et al. (2011) ^23^ | * | - | * | * | * | * | Moderate |
| Avlund, K., Holm-Pedersen, P., Morse, D.E. et al. (2005) ^24^ | - | - | ** | ** | * | * | Moderate |
| Avulund, K., Holm-Pedersen, P. and Schroll, M. (2001) ^25^ | * | - | ** | * | ** | * | Low |
| Brothwell, D., Jay, M., and Schonwetter, D. (2008) ^27^ | * | - | ** | * | * | * | Moderate |
| Castrejon-Perez, R.C., Borges-Yanez, S.A., Irigoyen-Camacho, M.E. and Cruz-Hervert, L.P. (2017) ^28^ | * | - | * | ** | ** | * | Low |
| Ganbavale, S.G., Papachristou, E., Mathers, J.C. et al. (2024) ^35^ | * | - | * | ** | ** | * | Low |
| Hassel, A., Koke, U., Schmitter, M., and Rammelsberg, P. (2006) ^38^ | * | - | * | ** | * | * | Moderate |
| Holm-Pedersen, P., Russell, S.L., Avlund, K. et al. (2006) ^41^ | - | - | ** | ** | * | * | Moderate |
| Hsu, K-J., Lee, H-E., Wu, Y-M. et al. (2013) ^42^ | - | - | ** | ** | * | * | Moderate |
| Kim, H-Y, Jang, M-S, Chung, C-P et al. (2009) ^52^ | - | - | * | ** | * | * | Moderate |
| Krustrup, U., Holm-Pedersen, P., Petersen, P.E. et al. (2008) ^55^ | - | - | ** | * | ** | * | Moderate |
| Lee, C-J, Ho, M-H, Joo, JY. et al. (2022) ^56^ | - | * | * | ** | * | * | Moderate |
| Liang, YH, Chou, C., Chen, YJ et al. (2020) ^57^ | - | * | * | * | * | * | Moderate |
| Lundgren M., Osterberg, T., Emilson, G. and Steen, B. (1995) ^59^ | * | - | * | * | * | * | Moderate |
| Musacchio, E., Perissinotto, E., Binotto, P. et al. (2007) ^60^ | * | - | ** | ** | * | * | Low |
| Niesten, D., Witter, D.J., Bronkhorst, E.M. and Creugers N.H.J. (2017)^61^ | * | - | ** | * | * | * | Moderate |
| Osterberg, T., Era, P., Gause-Nilsson, I., and Steen, B. (1995) ^62^ | * | - | * | * | * | * | Moderate |
| Rocha-Ortiz, J.A., Tepox-Puga, S.M., Borges-Yanez, S.A. et al. (2023) ^69^ | - | * | * | ** | * | * | Moderate |
| Tennstedt, S.L., Brambilla, D.L., Jette, A.M. and McGuire S.M. (1994) ^78^ | * | - | ** | ** | * | * | Low |
| Thorstensson, H. and Johansson, B. (2010) ^79^ | - | - | ** | ** | * | * | Moderate |
| Tsakos, G., Sheiham, A., Iliffe, S. et al. (2009) ^80^ | - | - | ** | ** | ** | * | Low |
| Vu, H., Vo, P. and Kim, H-D (2022) ^82^ | * | - | * | ** | ** | * | Low |
| Wu, L.L., Cheung, K.Y., Lam, P.Y.P., and Gao, X.L. (2017) ^84^ | - | * | ** | ** | ** | * | Low |
| Yanagisawa, S., Sugisawa, H., Harada, K. and Sugihara, Y. (2023) ^85^ | - | - | ** | * | ** | * | Moderate |
| Yang, H., Han, R. and Wang, Z. (2022) ^86^ | * | - | ** | * | ** | * | Low |
| Zini, A. and Sgan-Cohen, H.D. (2007) ^87^ | - | * | * | ** | ** | * | Low |
| Zini, A., Vered, Y. and Sgan-Cohen, H.D. (2011) ^88^ | - | * | ** | * | * | * | Moderate |
| Zulaga, D., Montoya, J., Contreras, C., and Herrera, R. (2012) ^89^ | - | - | * | ** | * | * | Moderate |
| Chan, C.C.K., Li, K.Y., Chen, H. et al. (2025) ^90^ | * | * | ** | * | ** | * | Low |

Table 3 – Risk of bias assessment for cross-sectional studies with prevalence data only using adapted NOS^22^ –

|  | Study sample selection | | Assessment of outcome |  |
| --- | --- | --- | --- | --- |
| Author (Year) | Representativeness of the study sample | Sample size | Assessment of the outcome(s) | Risk of bias |
| Aida, J., Kuriyama, S., Ohmori-Matsuda, K. et al. (2011) ^23^ | * | * | * | Low |
| Avlund, K., Holm-Pedersen, P., Morse, D.E. et al. (2005) ^24^ | * | - | * | Moderate |
| Avulund, K., Holm-Pedersen, P. and Schroll, M. (2001) ^25^ | * | - | * | Moderate |
| Brothwell, D., Jay, M., and Schonwetter, D. (2008) ^27^ | * | - | * | Moderate |
| Castrejon-Perez, R.C., Borges-Yanez, S.A., Irigoyen-Camacho, M.E. and Cruz-Hervert, L.P. (2017) ^28^ | * | - | ** | Low |
| Cocco, F., Campus, G., Strohmenger, L. et al. (2018) ^29^ | * | * | * | Low |
| Dai M., Song, Q., Lin, T. et al. (2023) ^30^ | * | * | * | Low |
| Dye, B.A., Weatherspoon, D.J. and Mitnik, G.L. (2019) ^31^ | * | - | * | Moderate |
| Edman, K., Ohrn, K., Nordstrom, B. and Holmlund, A. (2016) ^32^ | * | - | * | Moderate |
| Evren, B.A., Uludamar, A., Iseri, U. and Ozkan, Y.K. (2011) ^33^ | * | - | * | Moderate |
| Ferreira, R.C., Magalhaes, C.S. and Moreira, A.N. (2008) ^34^ | * | * | * | Low |
| Ganbavale, S.G., Papachristou, E., Mathers, J.C. et al. (2024) ^35^ | * | - | ** | Low |
| Go, H., Kim, E-Y., Jung, H-I. et al. (2022) ^36^ | * | - | * | Moderate |
| Griffin, S., Jones, J., Brunson, D. et al. (2012) ^37^ | * | - | * | Moderate |
| Hassel, A., Koke, U., Schmitter, M., and Rammelsberg, P. (2006) ^38^ | * | - | ** | Low |
| Hatta K., Ikebe, K., Gondo, Y. et al. (2018) ^39^ | * | - | * | Moderate |
| Heegard, K., Avlund, K., Holm-Pedersen et al. (2011) ^40^ | * | - | * | Moderate |
| Holm-Pedersen, P., Russell, S.L., Avlund, K. et al. (2006) ^41^ | * | - | * | Moderate |
| Hsu, K-J., Lee, H-E., Wu, Y-M. et al. (2013) ^42^ | * | - | ** | Low |
| Hunt, R., Beck, J., Lemke, J. et al. (1985) ^43^ | * | - | * | Moderate |
| Inomata, C., Ikebe, K., Okubo, H. et al. (2016) ^44^ | * | - | * | Moderate |
| Iosif, L., Preoteasa, C.T., Preoteasa E. et al. (2021) ^45^ | - | - | ** | Moderate |
| Islas-Granillo, H., Borges-Yanez, A., Medina-Solis, C. et al. (2016) ^46^ | * | * | * | Low |
| Islas-Granillo, H., Borges-Yanez, S.A., Medina-Solis, C.E. et al. (2014) ^47^ | - | - | ** | Moderate |
| Iwasaki, M., Yoshihara, A., Sato, M. et al. (2018) ^48^ | * | - | ** | Low |
| Janssens, B., Vanobbergen, J., Petrovic, M. et al. (2017) ^49^ | * | - | ** | Low |
| Jensen, P., Saunders, R., Thierer, T. and Friedman, B. (2008) ^50^ | * | - | ** | Low |
| Kim, E-K., Jung, Y-S., Kim, K-R. et al. (2018) ^51^ | - | - | ** | Moderate |
| Kim, H-Y, Jang, M-S, Chung, C-P et al. (2009) ^52^ | * | - | ** | Low |
| Komiyama, T., Ohi T., Miyoshi, Y. et al. (2016) ^53^ | * | - | * | Moderate |
| Komulainen, K., Ylostalo, P., Syrjala, A. et al. (2012) ^54^ | * | - | * | Moderate |
| Krustrup, U., Holm-Pedersen, P., Petersen, P.E. et al. (2008) ^55^ | * | - | ** | Low |
| Lee, C-J, Ho, M-H, Joo, JY. et al. (2022) ^56^ | * | - | * | Moderate |
| Liang, YH, Chou, C., Chen, YJ et al. (2020) ^57^ | - | - | ** | Moderate |
| Ling G.Y., Love, R.M., MacFadyen, E.E and Thomson, W.M. (2014) ^58^ | - | - | * | High |
| Lundgren M., Osterberg, T., Emilson, G. and Steen, B. (1995) ^59^ | * | - | * | Moderate |
| Musacchio, E., Perissinotto, E., Binotto, P. et al. (2007) ^60^ | * | - | * | Moderate |
| Niesten, D., Witter, D.J., Bronkhorst, E.M. and Creugers N.H.J. (2017) ^61^ | * | * | ** | Low |
| Osterberg, T., Era, P., Gause-Nilsson, I., and Steen, B. (1995) ^62^ | * | - | * | Moderate |
| Paulander J., Axelsson, P., and Lindhe, J. (2003) ^63^ | * | - | ** | Low |
| Phipps, K.R., Chan, B.K.S., Jennings-Holt, M. et al. (2009) ^64^ | * | - | ** | Low |
| Ramsay, S., Whincup, P., Watt, R. et al. (2015) ^66^ | * | - | ** | Low |
| Rapp, L., Sourdet, S., Vellas, B. and Lacoste-Ferre, M.H. (2017) ^67^ | - | - | * | High |
| Rise, J., and Holst, D. (1982) ^68^ | * | - | * | Moderate |
| Rocha-Ortiz, J.A., Tepox-Puga, S.M., Borges-Yanez, S.A. et al. (2023) ^69^ | * | * | * | Low |
| Sato, M., Iwasaki, M., Yoshihara, A., and Miyazaki, H. (2016) ^70^ | * | - | * | Moderate |
| Shimamura, M., Matsuyama, Y., Morita, A., and Fujiwara, T. (2022) ^71^ | * | - | ** | Low |
| Silva, M., Hopcraft, M., and Morgan, M. (2014) ^72^ | * | - | ** | Low |
| Singhal, A., Alofi, A., Garcia, R. and Sabik, L. (2021) ^73^ | * | - | * | Moderate |
| Siukosaari, P., Ainamo, A., and Narhi, T.O. (2005) ^74^ | * | - | * | Moderate |
| Siukosaari, P., Ajwani, S., Ainamo, A. et al. (2012) ^75^ | - | - | ** | Moderate |
| Syrjala, A-M. H., Raatikainen, L., Komulainen, K. et al. (2011) ^76^ | * | - | * | Moderate |
| Syrjala, A-M. H., Ylostalo, P., Hartikainen, S. et al. (2010) ^77^ | * | - | ** | Low |
| Tennstedt, S.L., Brambilla, D.L., Jette, A.M. and McGuire S.M. (1994) ^78^ | * | - | * | Moderate |
| Thorstensson, H. and Johansson, B. (2010) ^79^ | * | - | * | Moderate |
| Tsakos, G., Sheiham, A., Iliffe, S. et al. (2009) ^80^ | * | * | ** | Low |
| Uludamar, A., Evren, B.A., Iseri, U. and Ozkan, Y.K. (2011) ^81^ | * | - | * | Moderate |
| Vu, H., Vo, P. and Kim, H-D (2022) ^82^ | * | - | ** | Low |
| Wright, FAC., Chu, SK-Y, Milledge, KL. et al. (2017) ^83^ | * | - | ** | Low |
| Wu, L.L., Cheung, K.Y., Lam, P.Y.P., and Gao, X.L. (2017) ^84^ | * | * | ** | Low |
| Yanagisawa, S., Sugisawa, H., Harada, K. and Sugihara, Y. (2023) ^85^ | * | - | * | Moderate |
| Yang, H., Han, R. and Wang, Z. (2022) ^86^ | * | - | ** | Low |
| Zini, A. and Sgan-Cohen, H.D. (2008) ^87^ | * | * | ** | Low |
| Zini, A., Vered, Y. and Sgan-Cohen, H.D. (2011) ^88^ | * | - | ** | Low |
| Zulaga, D., Montoya, J., Contreras, C., and Herrera, R. (2012) ^89^ | * | 0 | ** | Low |
| Chan, C.C.K., Li, K.Y., Chen, H. et al. (2025) ^90^ | * | * | * | Low |
